# Natural products as modulators of age-associated gut microbiota dysbiosis: a systematic review of animal models

**DOI:** 10.3389/fphar.2026.1809547

**Published:** 2026-08-21

**Authors:** Siti Liyana Saud Gany, Nur Fatin Nabilah Mohd Sahardi, Noradliyanti Rusli, Anis Syauqina Mohd Zaffarin, Azraul Mumtazah Razak, Suzana Makpol

**Affiliations:** 1 Biochemistry Department, Faculty of Medicine, Universiti Kebangsaan Malaysia, Kuala Lumpur, Malaysia; 2 Secretariat of Research & Innovation, Faculty of Medicine, Universiti Kebangsaan Malaysia, Kuala Lumpur, Malaysia; 3 Faculty of Bioeconomic Food and Health Sciences, Universiti Geomatika Malaysia, Kuala Lumpur, Malaysia; 4 Faculty of Health Sciences, MAIWP International University, Kuala Lumpur, Malaysia; 5 Ageing and Degenerative Diseases Research Group, Universiti Kebangsaan Malaysia, Selangor, Malaysia

**Keywords:** ageing models, gut microbiota, healthy ageing, microbial diversity, microbiome modulation, microbiota-gut-brain axis, natural products, prebiotics

## Abstract

Ageing is increasingly recognised as a microbiota-driven process, with dysbiosis contributing to oxidative stress, chronic inflammation, and cognitive and functional decline. Natural products, long valued in traditional medicine, are emerging as targeted modulators of the gut ecosystem. This systematic review synthesised current evidence on natural product interventions in ageing animal models. Searches of four databases (2000–2024) yielded 762 records, of which 17 studies met eligibility criteria, encompassing naturally aged rodents, D-galactose-induced, genetically accelerated (SAMP8, *ob/ob*), and diet-induced models. Interventions spanned plant-derived compounds, probiotics, prebiotics, marine microalgae, and animal-derived products. Across models, natural products frequently normalized microbial communities, lowering the Firmicutes/Bacteroidetes ratio, suppressing pro-inflammatory taxa (*Helicobacter*, *Desulfovibrio*), and enriching beneficial genera (*Lactobacillus*, *Bifidobacterium*, *Akkermansia*). These shifts are linked to improved systemic outcomes, including enhanced antioxidant defences (SOD, GSH-Px, CAT), reduced levels of inflammatory mediators (IL-6, TNF-α, COX-2), and gains in memory, learning, and motor performance. Taken together, these findings demonstrate that natural products exert multi-targeted benefits in ageing by restoring microbial balance and attenuating hallmarks of decline. Yet, wide heterogeneity in animal models, intervention types, and outcome measures limits comparability. Standardised, long-term, and multi-omics approaches are urgently needed to establish mechanistic pathways and accelerate translation into human ageing interventions.

**Systematic Review Registration:**

https://www.crd.york.ac.uk/PROSPERO/view/CRD420250602174.

## 1 Introduction

The gut microbiota (GM) is a diverse community of bacteria, archaea, viruses, and fungi residing in the gastrointestinal (GI) tract. Far from being passive inhabitants, these microorganisms form a dynamic symbiotic relationship with the host, profoundly influencing physiological functions and overall health (Sankar et al., 2015; Han et al., 2021). Each harbours a unique microbial composition, which is estimated at around 100 trillion organisms, and is shaped by diet, lifestyle, and environmental factors, and is as distinctive as a fingerprint (Thursby and Juge, 2017; Scepanovic et al., 2019). Disruptions to this microbial equilibrium can lead to intestinal dysbiosis (ID), a state associated with a wide range of health conditions, including metabolic, immunological, cardiovascular, respiratory and neurodegenerative disorders (De Luca and Shoenfeld, 2018; Romano et al., 2021; Yeoh et al., 2021). Increasing evidence also links intestinal dysbiosis with the ageing process itself, positioning the gut microbiota as both a mediator and a potential biomarker of age-related decline (Nagpal et al., 2018).

Age-related gut dysbiosis is marked by a decline in microbial diversity and alterations in community composition, characterized by an increased abundance of potentially harmful microorganisms and a reduction in beneficial commensal bacteria (Conway and A Duggal, 2021). Ageing is also associated with changes in major bacterial phyla, particularly Firmicutes and Bacteroidetes, alongside a decrease in short-chain fatty acids (SCFA)-producing microbes that are essential for maintaining gut barrier function, immune regulation, and metabolic balance (Gyriki et al., 2025; Li et al., 2024). These microbial disturbances are often associated with increased intestinal permeability and a state of chronic, low-grade inflammation known as “inflammaging.” (McMahan et al., 2023). By influencing immune responses and generating pro-inflammatory metabolites, dysbiosis may perpetuate inflammatory processes that contribute to age-related decline (Conway and A Duggal, 2021). Notably, the relations between ageing and gut dysbiosis are considered bidirectional, where ageing-related physiological changes can alter the gut microbial environment (Tseng and Wu, 2025; Jayaraman and Pettersson, 2022). In contrast, dysbiosis can further exacerbate oxidative stress, inflammation, and other ageing-associated abnormalities (Liu et al., 2025; Zhang et al., 2025; Abahussin et al., 2025). The gut microbiota also exerts systemic effects through the gut-brain axis. Age-related dysbiosis may promote the release of microbial-derived lipopolysaccharides (LPS) and other inflammatory mediators that activate immune responses and neuroinflammatory pathways, linking intestinal dysfunction with cognitive impairment and neurodegenerative processes (Kim et al., 2016; Engler-Chiurazzi et al., 2023; Gupta and Kumar, 2026). Consequently, strategies aimed at restoring a healthy gut microbial ecosystem may offer a promising approach to support healthy ageing.

Ageing, meanwhile, is a complex process shaped by genetic, environmental, and stochastic factors that collectively influence lifespan and disease susceptibility (Rogina et al., 2000; Cevenini et al., 2008). Relatives of long-lived individuals often enjoy extended lifespans and lower risks of age-related diseases, suggesting that ageing may be modifiable and a promising target for intervention (Terry et al., 2004; Bucci et al., 2016). At the gastrointestinal level, ageing is accompanied by reduced motility, increased permeability, and changes in the enteric nervous system, all of which are closely associated with shifts in gut microbial composition (Claesson et al., 2011; Soenen et al., 2016). Among the various strategies explored to modulate gut microbiota and counteract age-related decline, natural products have attracted increasing attention due to their broad pharmacological properties and long history of use in traditional medicine.

Natural products are naturally occurring small molecules produced by living organisms, encompassing both primary and secondary metabolites (Abdel-Hafez et al., 1999). In the context of this review, natural products refer to naturally derived interventions, including plant-derived compounds, probiotics, marine-derived products, and animal-derived products, that can modulate gut microbiota composition and function. Despite their diverse origins, these interventions are grouped based on their shared capacity to influence host-microbiota interactions and ageing-related outcomes. Due to their structural complexity and prolonged retention within the GI tract, natural products readily interact with gut microbiota. Typically, exogenous substances remain in the small intestine for 1–6 h and persist in the colon for 1–3 days, providing ample opportunity for such interactions (Chu and Traverso, 2022). Although ageing is well known to be accompanied by gut microbiota disturbances (Odamaki et al., 2016; Fransen et al., 2017) the extent to which natural products can modulate these changes remains unclear. To our knowledge, no systematic review has comprehensively evaluated the effects of natural products on gut microbiota composition and associated functional outcomes in ageing animal models. Existing evidence is scattered across different ageing models and intervention types, limiting a clear understanding of common microbiota-related mechanisms and physiological effects. Therefore, this systematic review aims to evaluate the current evidence on the effects of natural products on gut microbiota composition and related functional outcomes in ageing animal models, to identify their potential role in mitigating age-associated physiological decline.

## 2 Materials and methods

This systematic review was conducted in accordance with the Preferred Reporting Items for Systematic Reviews and Meta-Analysis (PRISMA) 2020 guidelines and followed the PICOS framework (population, intervention, comparison, outcomes, and study design). Our literature review was registered in the international prospective register of systematic reviews, PROSPERO (https://www.crd.york.ac.uk/prospero/(accessed on 16 October 2024)), with registration number CRD420250602174.

### 2.1 Search strategy

A systematic search was conducted from 16th to 18 October 2024 on four electronic databases: Web of Science, Ovid MEDLINE, PubMed and Scopus. Search strategies for different databases were discussed among all authors using appropriate MeSH terms and keywords, as outlined in Table 1. The complete search strategies for each database are presented in Supplementary Table 1.

**TABLE 1:** Search strategies with appropriate MeSH terms were used for the literature search.

Search Line (concept)	Search strategies
Line 1 (Population)	Elderly OR aged
Line 2 (Population)	Gut microbiota OR gut microbiome* OR gastrointestinal microbiome* OR gut flora OR gut microflora OR gut microbiota OR intestinal micro* OR gastric microbiome*
Line 3 (Intervention)	Natural product* OR biologic* product* OR medicinal plant* OR medicinal herb* OR pharmaceutical plant*
Line 4 (Study)	Animal stud* OR animal model*

### 2.2 Eligibility criteria

The eligibility criteria for this study were formulated using the PICOS (Population, Intervention, Comparisons, Outcomes, Study) framework: (1) studies employing ageing rodent models (population); (2) studies evaluating naturally derived interventions, including plant-derived compounds, probiotics, prebiotics, marine-derived products, and other natural bioactive substance on gut microbiota in ageing rodents (intervention); (3) studies that have control groups or without interventions (comparisons); (4) studies that report at least one ageing-related outcome, including oxidative stress marker, inflammatory markers, DNA damage, cognitive function, muscle function or strength and composition of gut microbiota (outcomes); and (5) only *in vivo* experimental studies (study design). Only studies fullfilling all PICOS criteria were included in the review.

Studies were excluded from the review if any of these following exclusion criteria are met: (1) *in vitro*, *ex-vivo* or clinical studies; (2) studies that utilize non-rodents model of ageing; (3) studies that evaluate interventions other than natural products; (4) studies that do not evaluate the gut microbiota composition in rodents; (5) studies that did not report at least one of the predefined outcomes; or (6) studies that do not include a control group. Additionally, non-primary studies, case reports, conference abstracts and proceedings papers, editorials, letters, comments to the editor, reviews, meta-analysis, and book chapters were excluded from the review. Only studies published in English between 2000 and 2024 were included. The year 2000 was selected as the baseline to align with the advent and widespread adoption of modern molecular and high-throughput sequencing technologies in microbiome research, ensuring methodological comparability across the synthesized literature. Furthermore, restriction to rodent models was applied to improve biological comparability across studies in relation to ageing physiology, gut microbiota composition, and intervention responses.

### 2.3 Studies selection and data extraction

All articles obtained from electronic databases were imported into and saved in the EndNote reference manager, and duplicate records were removed. The remaining records were initially screened through the title and abstract by five reviewers (SLSG, NFNMS, NR, ASMZ and AMR) to identify studies that fulfilled the predefined eligibility criteria. Subsequently, full-text eligibility assessment was conducted independently by two reviewers. Inter-reviewers agreement during the full-text screening stage was assessed using Cohen’s kappa coefficient. Studies identified to have any of the exclusion criteria during the title, abstract, and full-text screenings were excluded. Relevant information, including rodent models used, age, type of intervention, duration of intervention and main findings) were extracted and tabulated using Microsoft Excel 365 MSO (Version 2407). Any disagreements and discrepancies during the screening and data extraction processes were resolved through discussions and consensus between all reviewers and consultation with SM.

### 2.4 Taxonomic validation of plant-derived natural products

The taxonomic validation of the plant-derived natural products included in this review was performed using Plants of the World Online (POWO; Royal Botanic Gardens, Kew). The botanical names, authorities, and family names were standardized according to accepted nomenclature. Detailed information regarding the plant parts used, preparation/extraction methods, and phytochemical characterizations was extracted from the eligible studies available, as outlined in the ConPhyMP reporting guideline (Supplementary Table 2). However, these details were inconsistently reported across the included studies and should therefore be considered when interpreting the overall evidence.

### 2.5 Quality assessment

**FIGURE 1:**
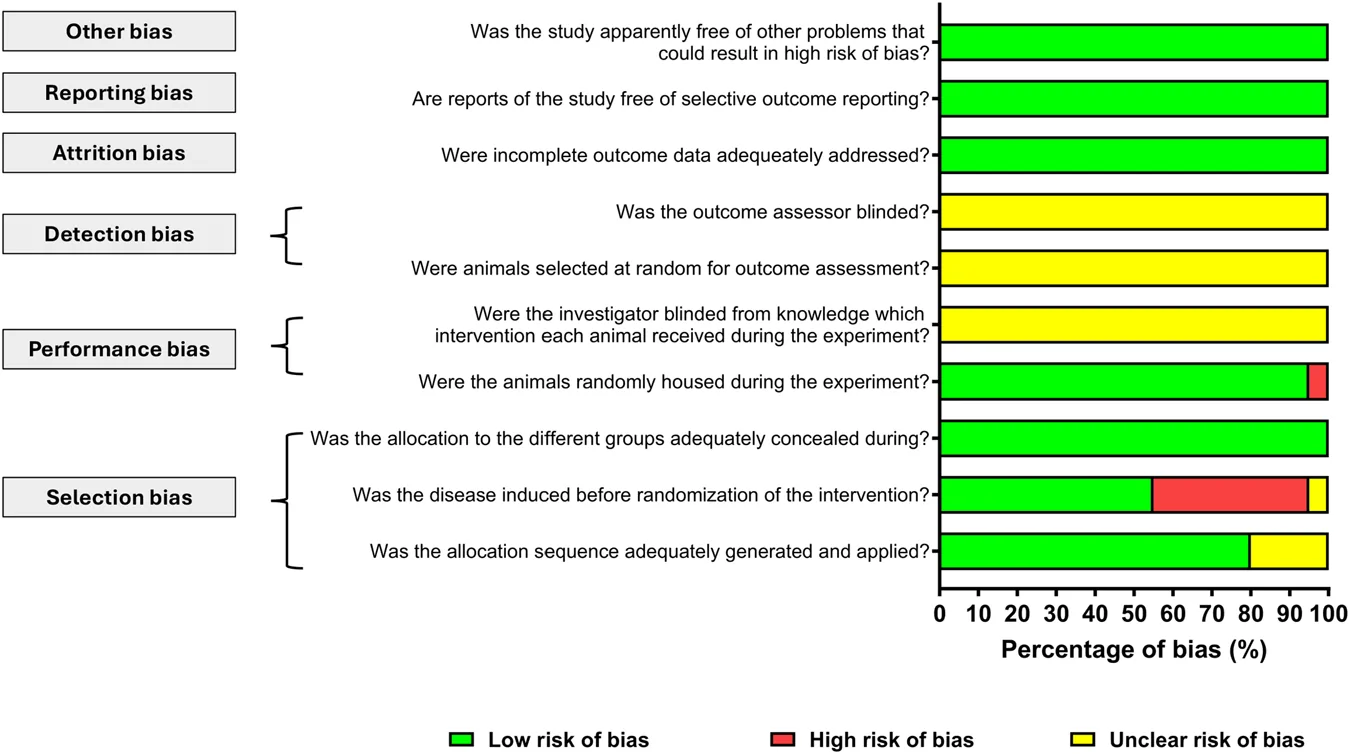
Results for the analysis of risk assessment using SYRCLE’s RoB tool.

To ensure the quality of articles included in this systematic review, a quality assessment was performed independently by two authors (ASMZ and NR) using SYRCLE’s risk of bias (RoB) tool for animal studies, following the CAMARADES checklist for bias risk assessment (Hooijmans et al., 2014). This tool allows for the systematic evaluation of the risk of bias across different domains–selection bias, performance bias, detection bias, attrition bias, reporting bias, and other biases (Figure 1). The results of the bias assessments were graded according to the item’s presence, where ‘Y’ indicates compliance, N′ indicates non-compliance, and ‘N/A’ indicates that data are unavailable.

The risk of bias was evaluated and reported strictly on a domain-by-domain basis. Any discrepancies between the two independent reviewers were resolved through comprehensive discussion until consensus was reached. All decisions made during the process, including points of disagreement and final consensus among assessors, were documented to provide a transparent record of the quality assessment process and support the reliability of the review’s findings.

Due to the exploratory nature of this review, findings were synthesized qualitatively to provide a comprehensive overview of natural product interventions targeting gut microbiota in ageing animal models and the associated outcomes evaluated across studies. Accordingly, a meta-analysis was not performed, as the objective was to summarize the available evidence rather than generate effect estimates. Consequently, formal statistical assessments of publication bias (such as funnel plots) and quantitative sensitivity analyses could not be methodologically performed. However, the inherent risk of publication bias favouring positive findings is acknowledged as a limitation of the available literature.

## 3 Results

### 3.1 Study selection

**FIGURE 2:**
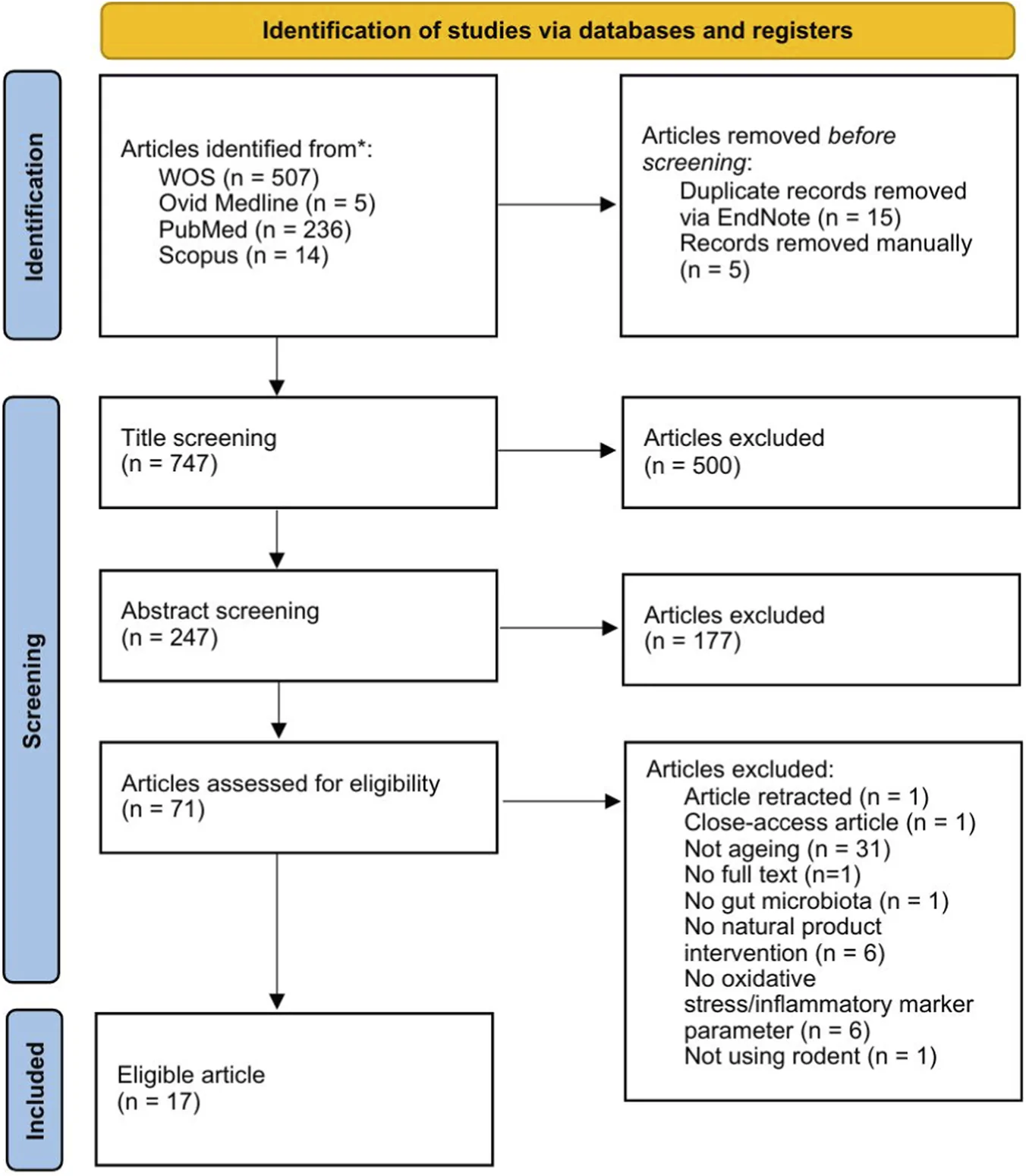
Flowchart for the identification and selection of studies according to the PRISMA statement.

From the initial database search, a total of 762 articles comprising of 507 articles from Web of Science, 236 articles from PubMed, 14 articles from Scopus, and 5 articles from Ovid Medline. Following the removal of 15 duplicates via EndNote and 5 articles manually removed, 742 articles remained for title screening. During title screening, 495 records were excluded, leaving 247 articles for abstract screening. Subsequently, 177 articles were excluded based on the predefined eligibility criteria, resulting in 70 full-text articles being assessed for eligibility. Inter-reviewer agreement during the full-text screening stage was assessed using Cohen’s kappa coefficient and demonstrated almost perfect agreement (Cohen’s κ = 0.87), with an overall agreement of 94.3%. Following full-text assessment, 53 articles were excluded due to various reasons, including articles retracted (n = 1), full-text article could be retrieved despite retrieval attempts (n = 2), not ageing-related (n = 27), no gut microbiota component (n = 1), no natural product intervention (n = 9), absence of oxidative stress or inflammatory marker outcomes (n = 6), studies not conducted using rodents (n = 1), review articles (n = 1) and other eligible criteria not met (n = 5). Finally, 17 articles fulfilled all inclusion criteria as mentioned in Section 2.2 and were included in this systematic review (Figure 2).

### 3.2 Description of the studies

Across the 17 included studies, the models varied widely, with the majority utilizing naturally aged rodents (∼41%), followed by D-galactose-induced models (∼29%), genetically accelerated models (∼24%) and diet-induced accelerated models (∼6%). A detailed breakdown of the experimental groups revealed a mean sample size of approximately 10 animals per group, with a median sample size of 9 animals per experimental group (overall ranging from 6 to 24 animals per group).

Despite established physiological differences in immune, metabolic, and microbial responses between males and females during ageing, sex differences were rarely analyzed as a primary outcome. The sole exception was Crowe et al., who reported isolated sex-specific microbial shifts (Crowe et al., 2020). They noted that the harmful expansion of *Enterobacteriaceae* occurred exclusively in old, high-calorie diet-fed male mice, which was subsequently reversed by their intervention. However, across the broader body of evidence, the systematic evaluation of sex-specific therapeutic responses remains a critical research gap. Details for the individual study characteristics are summarized in Table 2.

**TABLE 2:** Study characteristics.

Author (year)	Animal model	Type of ageing	Total no. of animals (age)	Category of natural product
Crowe et al. (2020)	Male and female C57BL/6J mice	Diet-induced accelerated ageing with HCD diet	Age: 9 weeks old N = 48 female N = 48 male	Animal-derived natural product (parasite nematode)
Fang et al. (2021)	SAMP8 mouse model (rapid ageing) SAMR1 mice as a normal ageing model	Genetically accelerated ageing models	Age:3 months old 1) Control (C) group, n = 10, SAMR1 mice; 2) SAMP8 model (M) group, n = 9 3) Low-dose probiotic (L) group, n = 10 4) High-dose probiotic (H) group, n = 10	Probiotic (microbial-derived natural product)
Gao et al. (2023)	C57BL/6 mice	Naturally ageing	1) Control group (6 weeks old), n = 8; 2) Aged group (40 weeks old), n = 8; 3) Aged + WHH1889 group (40 weeks old), n = 8	Probiotics (microbial-derived natural product)
He et al. (2019)	Kunming male mice (SPF level)	D-galactose-induced ageing	N = 50, 8 weeks old N = 10, 15 months old 1) Natural ageing control group (AC), n = 10 (15 months old) 2) Normal control group (NC): equal volume of 0.9% physiological saline, n = 10 (8 weeks old) 3) D-galactose induced ageing group (DG): D-galactose (500 mg/kg/day) + 0.9% physiological saline (equal volume), n = 10 (8 weeks old) 4) LMRPs: D-galactose (500 mg/kg/day) + MRPs (200 mg/kg/day), n = 10 (8 weeks old) 5) MMRPs: D-galactose (500 mg/kg/day) + MRPs (400 mg/kg/day), n = 10 (8 weeks old) 6) HMRPs: D-galactose (500 mg/kg/day) + MRPs (800 mg/kg/day), n = 10 (8 weeks old)	Plant-derived natural product
Arnold et al. (2021)	Female C57BL/6J SPF mice	Naturally ageing	n = 24, 6 w/o (young) n = 24, 60 w/o (old) 1) Young group: control diet + non-ABX (n = 6) 2) Old group: control diet + non-ABX (n = 6) 3) Young group: control diet + ABX (n = 6) 4) Old group: control diet + ABX (n = 6) 5) Young group: GOS diet + non-ABX (n = 6) 6) Old group: GOS diet + non-ABX (n = 6) 7) Young group: GOS diet + ABX (n = 6) 8) Old group: GOS diet + ABX (n = 6)	Prebiotic
Liu et al. (2019)	Male Sprague-Dawley rats	D-galactose induced ageing	N = 30 (6–8 weeks old) 1) Control group, n = 10 2) Recovery group, n = 10: Loperamide Hydrochloride (1.5 mg/kg) + D-galactose (125 mg/kg) for 4 weeks 3) ZYD group, n = 10: Loperamide Hydrochloride (1.5 mg/kg) + D-galactose (125 mg/kg) for 4 weeks	Plant-derived natural product (Traditional Chinese Medicine)
Ren et al. (2022)	C57BL/6J (SPF, male)	Naturally aged mice	N = 24 (18 months old) 1) Control group (CK), n = 6 2) Arenga pinnata flour, APF group, n = 6 3) Arenga pinnata starch, APS group, n = 6 4) Arenga pinnata retrograded starch, APRS group, n = 6)	Plant-based product
Shenghua et al. (2020)	Free SPF C57BL/6J female mice	Naturally aged mice	N = 18; n = 12, fed for 20 months to obtain natural ageing 1) Model group, n = 6 2) FTZ group, n = 6 3) Model group, n = 6 (2 months old)	Plant-derived natural product (Traditional Chinese Medicine)
Xiang et al. (2023)	SPF-grade Kunming (KM) male mice	D-galactose induced mice	N = 60 (6–8 weeks) 1) D-galactose group (ageing model control), n = 15 2) CK group (blank control group), n = 15 3) C1 group (low concentration of CBE group), n = 15 4) C2 group (hight concentration of CBE group), n = 15	Plant-derived natural product (Flower extract)
Xie et al. (2020)	ICR male mice	200 mg/kg/day D-galactosidase induced for 6 weeks	N = 60, (5 weeks old) 1) Normal control (NC) group, n = 10 2) Model group, n = 10 3) Positive group (VE), n = 10 4) Low-dose SCML (L-SCML) group, n = 10 5) Mid-dose SCML (M-SCML) group, n = 10 6) High-dose SCML (H-SCML) group, n = 10	Animal-derived natural product (larvae)
Yang et al. (2020)	ICR male mice	Naturally aged mice	N = 32 (14 months old) 1) Control group (CON), n = 8 2) Exogenous dietary advanced glycation end products (AGEs) diet group, n = 8 3) Quercetin group (Q), n = 8 4) AGE diet supplemented with quercetin group (AQ), n = 8	Probiotics isolated from feces of elderly people
Yu et al. (2023)	Female BALB/c mice	Naturally aged mice	N = 30 (4 months old) and N = 30 (20 months old) 1) Old mice (n = 10): FLPL0302 2) Old mice (n = 10): FLPL05 3) Old mice (n = 10): Control group 4) Young mice (n = 10): Control group 5) Young mice (n = 10): FLPL0302 6) Young mice (n = 10): FLPL05	Probiotics isolated from feces of elderly people
Zhang et al. (2021b)	Male SAMP8 mice and male senescence accelerated mouse resistant 1 (SAMR1)	Genetically accelerated ageing models	N = 60 (3–4 months old) 1) Standard diet (SD) group, n = 20 2) Wushen (WS) group, n = 20 3) Fecal microbiota transplantation (FMT) group, n = 20	Plant-derived natural product
Zhang et al. (2024)	Kunming (KM) mice	D-galactose induced ageing	N = 40 (8 weeks old) 1) Control group (C), n = 8 2) Model group (M), n = 8: 200 mg/kg/day D-gal 3) Astaxanthin group (M + A), n = 8: 200 mg/kg/day D-gal +10 mg/kg/day astanxanthin 4) H. pluvialis group (M + H), n = 8: 200 mg/kg/day D-gal + H. pluvialis 5) H. pluvialis residue group (M + HR), n = 8: 200 mg/kg/day D-gal + H. pluvialis residue	Microalgal natural product (marine microalgae)
Chen et al. (2024a)	Male BTBR WT and ob/ob mice	Naturally aged (BTBR WT) and genetically accelerated ageing models (ob/ob)	N = 32 (8 weeks old) 1) BTBR WT group, n = 8 2) BTBR WT + TSF group, n = 8 • Ob/ob group, n = 8 • Ob/ob + TSF group, n = 8	Plant-derived natural product (Traditional Chinese Medicine)
Gao et al. (2024)	Male SAMP8 and Senescence Accelerated Resistant Mouse (SAMR1)	Genetically accelerated ageing models	4 months old 1) SAMR1 group, n = N/A 2) SAMP8 ageing group, n = 6 3) SAMP8 Rg2 = 20 mg/kg, group, n = 6	Plant-derived natural product (Traditional Chinese Medicine)
Chen et al. (2024b)	Male C57BL/6J mice	Naturally aged mice	1) Young control, 12 weeks old (YC), n = 8 2) Old control, 18 months old (OC), n = 8 3) Royal jelly + Old, 18 months old (RJ), n = 8	Animal-derived functional food + bee product

Abbreviations: w/o, weeks old; m/o, months old; SAMP8, Senescence-Accelerated Moused Prone 8; SAMR1, Senescence-Accelerated Mouse Resistant 1; KM, kunming mice; SPF, specific pathogen free; RSGB, renshen guben; LMRPs, Low-dose Maillard Reaction Products; MMRPs, Medium-dose Maillard Reaction Products; HMRPs, High-dose Maillard Reaction Products; GOS, galacto-oligosaccharides; ABX, antibiotics; FTZ, fufang zhenzhu tiaozhi; CBE, guanshan cherry blossom extract; ICR, institute cancer research mice; SCML, Se-enriched *C. megacephala* larvae; WS, wushen; WT, wild type.

In terms of outcome evaluation, all 17 studies designated gut microbiota modulation as a primary endpoint, documenting significant shifts in microbial composition, or functional capacity following natural product interventions. Beyond microbial profiling, immunological and redox markers were the most frequently paired assessments. Inflammatory parameters were evaluated in 82.4% of the literature (14 studies), whereas oxidative stress markers were assessed in 35.3% of the studies (6 studies). In contrast, functional and genomic outcomes were notably underrepresented. Behavioral and cognitive function tests such as the Barnes maze and novel object recognition were included in only 29.4% of the studies (5 studies) and these were primarily restricted to research utilizing senescence-prone or diet-induced ageing models. Even so, despite its central role in the biology of ageing, none of the included studies evaluated DNA damage. Collectively, this distribution (summarized in Table 3) reveals a strong research emphasis on the microbiota-inflammatory axis, while critical functional endpoints and markers of genomic stability remain significant gaps in the current literature.

**TABLE 3:** Outcome parameters of the selected articles.

Author (year)	Gut microbiota	Oxidative stress	Inflammatory markers	DNA damage	Behavioral tests/Cognitive function
Crowe et al. (2020)	+	-	+	-	+
Fang et al. (2021)	+	-	+	-	+
Gao et al. (2023)	+	-	+	-	+
He et al. (2019)	+	+	+	-	-
Arnold et al. (2021)	+	-	+	-	-
Liu et al. (2019)	+	+	-	-	-
Ren et al. (2022)	+	-	+	-	-
Shenghua et al. (2020)	+	-	+	-	-
Xiang et al. (2023)	+	-	-	-	-
Xie et al. (2020)	+	+	-	-	-
Yang et al. (2020)	+	-	+	-	+
Yu et al. (2023)	+	-	+	-	-
Zhang et al. (2021a)	+	+	+	-	+
Zhang et al. (2024)	+	+	+	-	-
Chen et al. (2024a)	+	-	+	-	-
Gao et al. (2024)	+	+	+	-	-
Chen et al. (2024b)	+	-	+	-	-

A comparison of experimental designs across the different animal models reveals distinct methodological patterns tailored to each model’s specific biological strengths. First, chemically induced D-galactose models were predominantly utilized to investigate redox imbalances, with four of the five studies assessing oxidative stress markers such as SOD and MDA. In stark contrast, none of the exclusively naturally aged models evaluated oxidative stress; instead, all seven of these studies focused heavily on immunological shifts, measuring inflammatory markers like IL-6 and TNF-α. Genetically accelerated models (such as SAMP8 and *ob/ob* mice) effectively bridged this gap, frequently assessing both oxidative stress and chronic inflammation concurrently to capture a broader systemic decline. Furthermore, behavioral and cognitive functional assessments such as the Barnes maze, Y-maze, or novel object recognition were most densely concentrated in studies using senescence-prone or diet-induced models, which are specifically suited to manifest rapid, distinct functional declines in memory and motor performance. Finally, a clear pattern emerged regarding intervention duration: therapies evaluated in D-galactose models were heavily skewed toward short-term applications (typically 4–6 weeks), whereas studies employing naturally aged or genetically accelerated rodents consistently utilized extended intervention periods (ranging from 12 weeks to 9 months) to better align with the gradual progression of age-related physiological and microbial shifts.

### 3.3 Effect of natural products on gut microbiota

Across the 17 included studies, natural product interventions consistently modulated gut microbiota composition at the phylum and genus levels. The evaluated interventions were predominantly plant-derived therapies (52.9%, 9 studies), encompassing Traditional Chinese Medicine, functional foods, and isolated compounds. The remaining studies utilized microbial probiotics (17.6%, 3 studies), animal-derived products (17.6%, 3 studies), prebiotics (5.9%, 1 study), and marine microalgae (5.9%, 1 study). Notably, overlap exists within experimental designs. Zhang, Chen et al. uniquely bridged intervention types by simultaneously testing a plant-derived food mixture (*Wushen*) alongside a Faecal Microbiota Transplantation (FMT) arm to directly compare dietary and microbiome-transfer therapies (Zhang J. et al., 2021). However, the direction and magnitude of these effects varied depending on the type of intervention and the ageing model.

A quantitative assessment of alpha diversity, explicitly reported in nine studies, yielded highly variable results rather than a consistent age-related decline or intervention-induced increase. Specifically, three studies reported a statistically significant increase in diversity following intervention (Xie et al., 2020; Yang et al., 2020; Chen D. Q. et al., 2024), while two studies reported a decrease (effectively normalizing an abnormal age-related spike in diversity) (Shenghua et al., 2020; Gao et al., 2024), and three studies observed no statistically significant changes in alpha-diversity between groups (Zhang J. et al., 2021; Chen D. Q. et al., 2024; Liu et al., 2019).

Similarly, the Firmicutes/Bacteriodetes (F/B) ratio did not shift unidirectionally across the literature. Of the ten studies detailing significant shifts in these specific phyla, five studies reported a decreased F/B ratio (or an enrichment of Bacteriodetes relative to Firmicutes) following natural product administration (Shenghua et al., 2020; Liu et al., 2019; Gao et al., 2023; Ren et al., 2022; Zhang et al., 2024). Conversely, five studies reported an increased F/B ratio; this was particularly evident in genetically accelerated models, where baseline ageing was paradoxically characterized by a depleted F/B ratio (Crowe et al., 2020; Chen D. Q. et al., 2024; Gao et al., 2024; He et al., 2019; Chen L. et al., 2024). This indicates that rather than uniformly lowering the F/B ratio, these interventions tend to normalize phylum-level distributions toward the baseline of healthy, non-aged controls.

Despite variability in broad diversity metrics, more consistent patterns emerged at the genus and family levels. Across the included studies, 10 of the 17 studies reported a significant suppression of potentially pathogenic or pro-inflammatory taxa, most notably *Helicobacter* and *Desulfovibrio* (Crowe et al., 2020; Xie et al., 2020; Chen D. Q. et al., 2024; Shenghua et al., 2020; Gao et al., 2024; Gao et al., 2023; Ren et al., 2022; He et al., 2019; Xiang et al., 2023; Yu et al., 2023). Concurrently, interventions frequently enriched beneficial SCFA-producing commensals; 11 studies documented statistically significant increases in taxa such as *Lactobacillus, Bifidobacterium*, and *Akkermansia* following treatment (Zhang J. et al., 2021; Xie et al., 2020; Chen D. Q. et al., 2024; Gao et al., 2024; Gao et al., 2023; Ren et al., 2022; He et al., 2019; Chen L. et al., 2024; Xiang et al., 2023; Arnold et al., 2021; Fang et al., 2021).

It is important to note that while the reported taxonomic shifts were statistically significant within their respective experimental designs (typically p < 0.05), none of the included studies systematically reported standardized effect sizes (e.g., Cohen’s d). Consequently, comparing the absolute magnitude of efficacy between different natural products remains methodologically limited. Nonetheless, the synthesized data (summarized in Table 4) suggests that natural products possess the capacity to reshape the gut ecosystem, primarily by mitigating model-specific dysbiosis and enriching commensal populations.

**TABLE 4:** Effect of natural product on gut microbiota.

Author (year)	Type of natural product, dosage, intervention duration	Method of detection	Gut microbiota effect (kingdom, phylum)
Crowe et al. (2020)	ES-62 derived from the filarial nematod *Acanthocheilonema vitae* Dose: 1 μg/week Duration: from 9 weeks old till death	*DNA extraction from ileum and colon faecal matter (QIAamp DNA Stool Mini Kit)* *↓* *Shotgun metagenomic sequencing (Ion Torrent PGM platform)* *↓* *Library preparation (NEBNext DNA fragmentation & IonXpress barcoding)* *↓* *Data analysis: MG-RAST*	• *Verrucomicrobioa* ↓ in ageing (chow-fed), in HCD-fed, also no effect with ES-62 • F/B ratio ↓ in old age (chow-fed) and ↑ in old ES-62 + HCD-fed mice • *Desulfovibrionaceae* (δ-Proteobacteria) ↑ in HCD-fed mice and ↓ in ES-62 + HCD-fed mice • *Enterobacteriaceae* (γ-Proteobacteria) ↑ in old HCD-fed male mice only and ↓ in ES-62 + HCD-fed male mice
Fang et al. (2021)	Probiotic combination (4 bacterial strains) 1. Lactobacillus fermentum SX-0718 2. Lactobacillus casei SX-1107 3. Bifidobacterium longum SX-1326 4. Bifidobacterium animalis SX-0582 Dose - Low-dose group (L): 1 × 10^7^ CFU/mL of each strain, daily - High-dose group (H): 1 × 10^9^ CFU/mL of each strain, daily *Duration: 18 weeks*	*Fecal DNA extraction (Qiagen Kit)* *↓* *16S rRNA gene sequencing (V4 region) on Illumina HiSeq2000* *↓* *Bioinformatics: Vsearch (OTU clustering)/QIIME2 DADA2 (ASV analysis)* *↓* *Analysis: species composition, alpha and beta diversity, differential species analysis*	• *Alistipes*: ↑ in ageing model (M) group; ↓ with high and low dose of probiotic combination group (L & H) • *Prevotella*: ↑ in M group; ↓ in L & H group • *Odoribacter*: ↑ in M group • *Lactobacillus*: ↑ in M group • *Oscillibacter*: ↑ in M group • *Alloprevotella*: ↓ in M group; ↑ in L & H group • *Barnesiella*: ↓ in M group • *Akkermansia*: ↓ in M group • *Acetatifactor*: ↑ in L & H group • *Clostridium XIVa*: ↑ in L & H group
Gao et al. (2023)	*L. helveticus WHH1889* Dose: 0.2 mL/day, 5 × 10^9^ CFU/mL *Duration: 12 weeks*	*Fecal DNA extraction (QIAamp DNA Stool Kit)* *↓* *16S rRNA gene sequencing (full-length) on PacBio Sequel II platform* *↓* *Bioinformatics: QIIME2 (DADA2, ASV analysis), taxonomy with SILVA database* *↓* *Analysis: microbial composition, diversity; visualization with R (ggplot2)*	Phylum • *Firmicutes*: ↑ in aged mice; ↓ in aged mice + WHH1889 group • *Desulfobacterota*: ↑ in aged mice; ↓ in aged mice + WHH1889 group • *Bacteroidota*: ↓in aged mice; ↑ in aged mice + WHH1889 group Genus • *Unclassified Muribaculaceae*: ↓in aged mice; ↑ in aged mice + WHH1889 group • *Lactobacillus*: ↓in aged mice; ↑ in aged mice + WHH1889 group • *Muribaculum*: ↓in aged mice; ↑ in aged mice + WHH1889 group • *Prevotellaceae_UCG_001*: ↓in aged mice; ↑ in aged mice + WHH1889 group • *Prevotellaceae_NK3B_31 group*: ↓in aged mice; ↑ in aged mice + WHH1889 group • *Lachnospiraceae_NK4 A136 group*: ↑ in aged mice; ↓ in aged mice + WHH1889 group • *Unclassified Clostridiales*: ↑ in aged mice; ↓ in aged mice + WHH1889 group • *Unclassified Desulfovibrionaceae*: ↑ in aged mice; ↓ in aged mice + WHH1889 group • *Mucispirillum*: ↑ in aged mice; ↓ in aged mice + WHH1889 group • *Oscillibacter*: ↑ in aged mice; ↓ in aged mice + WHH1889 group • *Roseburia*: ↑ in aged mice; ↓ in aged mice + WHH1889 group • *Alloprevotella*: ↑ in aged mice; ↓ in aged mice + WHH1889 group • *Colidextribacter*: ↑ in aged mice; ↓ in aged mice + WHH1889 group • *Desulfovibrio*: ↑ in aged mice; ↓ in aged mice + WHH1889 group • *Unclassified Firmicutes*: ↑ in aged mice; ↓ in aged mice + WHH1889 group • *Rikenella*: ↑ in aged mice; ↓ in aged mice + WHH1889 group • *Unclassified Peptococcaceae*: ↑ in aged mice; ↓ in aged mice + WHH1889 group • *Anaerotruncus*: ↑ in aged mice; ↓ in aged mice + WHH1889 group
He et al. (2019)	Rapeseed peptide Maillard reaction products (MRPs): Dose: - Low-dose: 400 mg/kg/day - Medium-dose: 500 mg/kg/day - High-dose: 800 mg/kg/day Duration: 6 weeks	*Fecal DNA extraction (E.Z.N.A. Stool DNA Kit)* *↓* *16S rRNA gene sequencing (V3-V4 region) on Illumina HiSeq2500* *↓* *Bioinformatics: QIIME (OTU clustering, taxonomy with SILVA & UNITE)* *↓* *Analysis: alpha diversity (MOTHUR, R), beta diversity (PCoA, NMDS, UPGMA, heatmap)*	Phylum • *Bacteroidetes*: ↑ in Ageing control (AC) and D-galactose-induced ageing (DG); ↓ in MRPs (low, medium, and high dose) groups • *Firmicutes*: ↓ in AC & DG group; ↑in MRPs (low, medium, and high dose) groups • *Proteobacteria*: ↑ in AC & DG group; ↓ in MRPs (low, medium, and high dose) groups Family • *Enterobacteriaceae*: ↑ in AC & DG group; ↓ in MRPs (low, medium, and high dose) groups • *Erysipelotrichaceae*: ↑ in AC & DG group; ↓ in MRPs (low, medium, and high dose) groups • *Desulfovibrionaceae*: ↑ in AC & DG group; ↓ in MRPs (low, medium, and high dose) groups • *Helicobacteraceae*: ↑ in AC & DG group; ↓ in MRPs (low, medium, and high dose) groups • *Bacteroidales_S24-7_group*: ↓ in AC & DG group; ↑in MRPs (low, medium, and high dose) groups • *Lachnospiraceae*: ↓ in AC & DG group; ↑in MRPs (low, medium, and high dose) groups • *Lactobacillaceae*: ↓ in AC & DG group; ↑in MRPs (low, medium, and high dose) groups • *Prevotellaceae*: ↓ in AC & DG group; ↑in MRPs (low, medium, and high dose) groups • *Rikenellaceae*: ↓ in AC & DG group; ↑in MRPs (low, medium, and high dose) groups • *Ruminococcaceae*: ↓ in AC & DG group; ↑in MRPs (low, medium, and high dose) groups Genus • *Helicobacter*: ↑ in AC & DG group; ↓ in MRPs (low, medium and high dose) groups • *Lactobacillus* spp.: ↓ in AC & DG group; ↑in MRPs (low, medium, and high dose) groups • *Bifidobacterium* spp.: ↓ in AC & DG group; ↑in MRPs (low, medium, and high dose) groups
Arnold et al. (2021)	Prebiotic galacto-oligosaccharides (GOS): Dose: replaced 71.8 g cellulose/kg with 71.8 g pure GOS Duration: 2 weeks	*DNA extraction from fecal samples (Qiagen DNeasy Stool Kit + bead-beating)* *↓* *16S rRNA sequencing (V4 region) on Illumina MiSeq* *Shotgun metagenomic sequencing also performed* *↓* *Bioinformatics: QIIME2 (cutadapt, DADA2, taxonomy with Greengenes)* *↓* *Analysis: alpha diversity (Faith PD, Shannon, observed species); beta diversity (UniFrac, Bray-Curtis, PCoA), ANCOM (differential abundance)*	Old – GOS diet •↑ *Akkermansia muciniphila* (β-galactosidase abundance) •↑ *Bacteroides thetaiotaomicron* (β-galactosidase abundance) •↑ *Bifidobacterium psudolongum* •↑ *Enterococcus faecalis* Young – GOS diet •↑ *Bifidobacterium pseudolongum* •↑ *Lactobacillus johnsonii* •↓ Undetermined metabolism taxa (T2) Antibiotic-treated (both ages, both diets) •↓ *Bifidobacterium* spp. •↓ *Lactobacillus* spp. • GOS-fed: ↑ *Saccharolytic taxa,* ↓ non-saccharolytic taxa Control-fed: ↑ non-saccharolytic taxa
Liu et al. (2019)	Zengye Decoction (ZYD) Dose: 63 mg/mL *Duration: 5 weeks*	*Fecal DNA extraction (chemical lysis with N-lauroyl sarcosine, Tris buffer, guanidine thiocyanate)* *↓* *16S rRNA gene sequencing (V4 region) on Illumina HiSeq 2500* *↓* *Bioinformatics: FLASH (read merging), UCHIME (chimera removal), QIIME (OTU clustering), taxonomy with Greengenes (RDP classifier, PyNAST alignment)* *↓* *Analysis: alpha diversity (Shannon, rarefaction), beta diversity (UniFrac, PCoA, clustering), LEfSe (differential taxa), PICRUSt (functional prediction, KEGG pathways), STAMP (functional profiling)*	ZYD vs. recovery (constipated) •↑ Bacteroidetes, Actinobacteria, Tenericutes after ZYD; ↓ Firmicutes after ZYD ZYD vs. Control No significant differences
Ren et al. (2022)	1) Control group (CK): basal diet 2) APF group: basal diet +10% *Arenga pinnata* flour, 3) APS group: basal diet +10% *Arenga pinnata* starch 4) APRS group: basal diet +10% *Arenga pinnata* retrograded starch *Duration: 8 weeks*	*Fecal DNA extraction* *↓* *16S rRNA sequencing* *↓* *Bioinformatics: MOTHUR (OTU clustering, alpha diversity), QIIME (PCoA, beta diversity)* *↓* *Analysis: Good’s coverage (sequencing depth), LEfSe (differential taxa, LDA > 4), Venn diagrams (R package VennDiagram)*	Phylum • APF: ↓ Firmicutes, ↑ Bacteroidetes, ↑ Actinobacteria • APS: ↑ Bacteroidetes, ↑ Actinobacteria • All groups: ↓ Proteobacteria Family • APF: ↑ Bifidobacteriaceae, ↑ Muribaculaceae, ↑ Prevotellaceae, ↓ Lachnospiraceae • APS: ↑ Bifidobacteriaceae, ↑ Muribaculaceae • APRS: ↑ Ruminococcaceae, ↑ Monoglobaceae, ↑ Saccharimonadeceae • All groups: ↓ Sutterellaceae Genus • APF: ↑ Prevotellaceae_UCG-001, ↑ Bifidobacterium • APS: ↑ Lachnospiraceae_NK4A136 (potential probiotic) • APRS: ↑ Ruminococcaceae_UCG-014, ↑ Eubacterium_xylanophilum, ↑ Roseburia, ↑ Monoglobus, ↑ Butyricicoccus, ↑ Lactobacillus
Shenghua et al. (2020)	Fufang Zhenzhu Tiao Zhi (FZT) Dose: 1.0 g/kg body weight *Duration: 12 weeks (daily)*	*Fecal DNA extraction (E.Z.N.A. Stool DNA Kit)* *↓* *16S rRNA gene sequencing (V3-V4 region) on Illumina GAIIx* *↓* *Bioinformatics: QIIME (quality filtering, OTU clustering with UCLUST), taxonomy with RDP classifier* *↓* *Analysis: alpha diversity (Shannon index), beta diversity (PCoA)*	• Diversity: ↑ aging mice; ↓ in FZT group Phylum • *Firmicutes*: ↑ in ageing mice; ↓ in FZT group • *Bacteroidetes*: ↓ *in* ageing mice; ↑ in FZT group • *Bacteroidetes/Firmicutes* ratio: ↓ in ageing mice Family • *Dehalobacteriaceae*: ↑ in ageing mice; ↓ in FZT group • *Peptococcaceae*: ↑ in ageing mice; ↓ in FZT group • *Turicibacteraceae*: ↑ in ageing mice; ↓ in FZT group • *S24-7 (Muribaculaceae)*: ↓ in ageing mice; partial restoration of S24-7 Genus • *Dehalobacterium*: ↑ in ageing mice; ↓ in FZT group • *Turicibacter*: ↑ in ageing mice; ↓ in FZT group • *Mucispirillum*: ↑ in ageing mice; ↓ in FZT group • *Bilophila*: ↑ in ageing mice; ↓ in FZT group • *Moryella*: ↑ in ageing mice; ↓ in FZT group • *Desulfovibrio*: ↑ in ageing mice; ↓ in FZT group • *Rikenella*: ↓ in ageing mice; ↑ in FZT group • *Alkaliphilus*: ↓ in ageing mice; ↑ in FZT group • *Haererehalobacter*: ↓ in ageing mice; ↑ in FZT group
Xiang et al. (2023)	Guanshan cherry blossom from *Crasus serrulate ‘Sekiyama’* 1) C1 group: 0.2 mL CBE (200 mg/kg/day) 2) C2 group: 0.2 mL CBE (400 mg/kg/day) *Duration: 6 weeks (daily)*	*DNA extraction (CTAB method)* *↓* *16S rRNA sequencing with QIIME2 (OTU clustering, diversity analysis)* *↓* *Functional prediction: PICRUSt (KEGG pathways)* *↓* *Statistical analysis: STAMP (group differences)*	Diversity: ↓ *Firmicutes* in D-gal ageing mice; ↑ some pathogenic taxa (*Desulfovibrio*) - CBE (C1, C2) reversed microbial community shifts towards control Phylum • *Firmicutes*: ↑ in C1 and C2 • *Verrucomicrobiota*: ↑ in C1 > D-gal • *Desulfovibrio*: ↓ in CBE groups Genus • *Akkermansia*: ↑ in C1 • *Desulfovibrio*: ↓ in CBE groups • *Helicobacter*: ↓ in CBE groups • *Clostridiales* unclassified: ↓ in CBE groups • *Candidatus_Saccharimonas*: ↓ in CBE groups
Xie et al. (2020)	Selenium-enriched *C. megacephala* larvae (SCML) 1) L-SCML group: 300 ug/kg/day SCML 2) M-SCML group: 1000 ug/kg/day SCML 3) - H-SCML group: 3,000 ug/kg/day SCML *Duration: 6 weeks (daily)*	*Fecal DNA extraction (BGI, Wuhan)* *↓* *16S rRNA gene sequencing (V3-V4 region) on Illumina HiSeq* *↓* *Bioinformatics: FLASH (read merging), USEARCH (OTU clustering), taxonomy with RDP classifier* *↓* *Analysis: microbial diversity and community structure (R software)*	Diversity: ↓ model group; ↑ in VE & H-SCML group Phylum • *Proteobacteria*: ↑ in model group, ↓ in VE and SCML groups • *Bacteroidetes*: ↓ in model group, ↑ in VE and SCML groups • *Firmicutes*: significant changes in VE, M-SCML and H-SCML groups • *Actinobacteris*: ↑ in M-SCML Genus • *Helicobacter*: ↑ in model group, ↓ in VE and SCML groups • *Clostridium*: ↑ in model group, ↓ in VE and SCML groups • *Ruminococcus*: ↓ in model group; no significant restoration in treatment groups • *Oscillospira*: ↑ in model group, ↓ in VE and SCML groups • *Bacteroides*: ↑ in VE & L-SCML groups • *Lactobacillus*: ↑ in M-SCML & H-SCML groups • *Prevotella*: ↑ in VE *Sutterella*: ↑ in M-SCML
Yang et al. (2020)	Quercetin (0.08%) *Duration: 21 days*	*DNA extraction from cecal content (PowerMax kit)* *↓* *16S rRNA gene sequencing (V4 region) on Illumina HiSeq 4,000* *↓* *Bioinformatics: QIIME (quality filtering, OTU clustering with Vsearch), taxonomy with SILVA128 database* *↓* *Analysis: alpha diversity (Chao1, ACE, Shannon, Simpson)*	Diversity: ↑ in AQ group Phylum • *Tenericutes*: ↑ in AGE group & AQ group • *Epsilonbacteraeota*: ↓ in Q group • *Verrucomicrobia*: ↓ in AQ group Genus • Ruminococcaceae_UCG-014: ↑ in AGE group • Lachnspiraceae_UCG-008: ↑ in AGE group • Balutia: ↓ in AGE and AQ group • Anaerotruncus: ↓ in AQ group
Yu et al. (2023)	1) 1 × 10^8^ CFU/d *L plantarum* FLPL0302 2) 1 × 10^8^ CFU/d *L. Plantarum* FLPL05 *Duration: 2 months*	*Fecal DNA extraction (MagPure Stool DNA KF Kit B)* *↓* *16S rRNA gene sequencing (V3-V4 region) on Illumina MiSeq* *↓* *Bioinformatics: FLASH (read merging), UPARSE (OTU clustering), taxonomy with RDP classifier (Greengenes, QIIME), USEARCH (abundance)* *↓* *Analysis: LEfSe (differential taxa)*	• Firmicutes: ↑ with L. plantarum FLPL05 and ↓ with L. plantarum FLPL0302 • Deferribacteres: ↑ with L. plantarum FLPL05 • Bacteroidetes: ↓ with L. plantarum FLPL05; ↑ with L. plantarum FLPL0302 • Teneribacteria: ↓ with L. plantarum FLPL05 and L. plantarum FLPL0302 • Enterobacter: ↑ with L. plantarum FLPL05 • Escherichia: ↑ with L. plantarum FLPL05 • Psychrobacter: ↑ with L. plantarum FLPL05 • Trabulsiella (Proteobacteria): ↑ with L. plantarum FLPL05 • LEfSe: notable differences in Lactobacillus and Desulfovibrio abundance
Zhang et al. (2021a)	*Wushen (WS)* Dose: ? Duration: N/A	*Fecal DNA extraction (QIAamp DNA Stool Mini Kit)* *↓* *16S rRNA gene sequencing (V3-V4 region) on Illumina MiSeq* *↓* *Bioinformatics: FLASH (read merging), USEARCH (OTU clustering), taxonomy with RDP classifier (SILVA database)* *↓* *Analysis: alpha diversity (Chao1), beta diversity (PCoA, PCA, UniFrac)*	• α-diversity: ↓ bacterial richness and abundance in SD group; no significance in other groups • *Prevotellaceae*: ↑ in WS group vs. SD group • *Ruminococcus*: ↑ in WS group vs. SD group • *Butyrivibrio*: ↑ in WS group vs. SD group • *Lachnoclostridium*: ↓ in WS group vs. SD group • *Ruminiclostridium*: ↓ in WS group vs. SD group • *Eubacterium coprostanoligenes*: ↓ in WS group vs. SD group • *Intestinimonas*: ↓ in WS group vs. SD group • *Clostridium* sp. *ASF356*: ↓ in WS group vs. SD group • *Lachnospiraceae*: ↓ in WS group vs. SD group
Zhang et al. (2024)	*Haematococcus pluvialis (H. pluvialis)* 1) M + A group: 10 mg/kg/day astaxanthin 2) M + H group: 1 g/kg/day *H. pluvialis* + 10 mg/kg/day astaxanthin 3) M + HR: 1 g/kg/day *H. pluvialis* *Duration: 10 weeks*	*Fecal DNA extraction* *↓* *16S rRNA gene sequencing (V3-V4 region)* *↓* *Analysis: online* on majorbio.com	• *Firmicutes*: ↑ in D-gal group; ↓ in M + A & M + HR • *Firmicutes/Bacteroidetes* (F/B ratio): ↑ in D-gal group; ↓ in M + A & M + HR • *Bacteroidetes*: ↓ in D-gal group; ↑ in M + A & M + HR • *Lactobacillus*: ↑ in D-gal group; ↓ in M + A, M + H & M + HR • *Lachnospiraceae*: ↓ in D-gal group; ↑ in M + A & M + H • *Stretpcoccus*: ↓ in D-gal group; ↑ in M + A & M + HR
Chen et al. (2024a)	Tangshen Formula (TSF) Dose: 2.4 g/kg/day *Duration: 24 weeks*	*Colon DNA extraction (PowerSoil DNA Isolation Kit)* *↓* *16S rRNA gene sequencing (V3-V4 region) on Illumina MiSeq PE300* *↓* *Bioinformatics: UPARSE (OTU clustering)* *↓* *Analysis: alpha diversity (Chao1, Shannon, Simpson via Dr. TOM, BGI), beta diversity (PLS-DA)*	α-diversity: • Species richness: ↑ in aged ob/ob vs. WT; ↓ after TSF • Diversity: ↓ in aged ob/ob vs. WT; partially restored by TSF Phylum • *Firmicutes*: ↓ in aged ob/ob • *Bacteroidetes*: ↑ in aged ob/ob • *Proteobacteria*: ↑ in aged ob/ob; ↓ with TSF group • *Firmicutes/Bacteroidetes (F/B)*: ↓ in aged ob/ob; ↑ with TSF group Genus • *Alistipes*: ↑ in aged ob/ob; ↓ with TSF • *Parabacteroides*: ↑ in aged ob/ob; ↓ with TSF • *Lactobacillus*: ↑ in aged ob/ob; ↓ with TSF • *Bacteroides*: ↑ in aged ob/ob; ↓ with TSF • *Alloprevotella*: ↑ in aged ob/ob; ↓ with TSF • *Lachnospiracea_incertae_sedis*: ↑ in aged ob/ob; ↓ with TSF • *Clostridium_XIVa*: ↓ in aged ob/ob; ↑ with TSF *Helicobacter*: ↓ in aged ob/ob; ↑ with TSF
Gao et al. (2024)	Rg2, panaxatriol type ginsenoside Dose: 20 mg/kg *Duration: 3 months (daily)*	*Fecal DNA extraction (QIAamp DNA Stool Kit)* *↓* *16S rRNA gene sequencing (V3-V4 region) with primers 338F/806R* *↓* *PCR amplification (ABI GeneAMP 9700), purification (AxyPrep kit), quantification (QuantiFluor-ST)*	• α-diversity: ↑ in SAMP-WT group; ↓ with Rg2 treatment Phylum • *Firmicutes*: ↑ in Rg2 treatment vs. SAMP8-WT • *Bacteroidota*: ↓ in Rg2 treatment vs. SAMP8-WT • *Firmicutes/Bacteroidota* (F/B ratio): ↓ in SAMP8-WT; ↑ in Rg2 treatment • *Proteobacteria*: no significance • *Actinomycetes*: no significance Genus • *Lactobacillus*: ↓ in SAMP8-WT group; ↑ with Rg2 treatment • *norank f_Muribaculaceae*: ↓ in SAMP8-WT group; ↑ with Rg2 treatment • *Staphylococcus*: ↑ in SAMP8-WT; ↓ with Rg2 treatment • *Lachnospiraceae_NK4A136_group*: ↑ in SAMP8-WT; ↓ with Rg2 treatment
Chen et al. (2024b)	Royal jelly (RJ) Dose: 1500 mg/kg/bw *Duration: 9 months*	*Fecal DNA extraction (CTAB/SDS method)* *↓* *16S rRNA gene sequencing (V3-V4 region) on Illumina NovaSeq 6000* *↓* *Bioinformatics: OTU clustering, taxonomy annotation* *↓* *Analysis: alpha diversity (Shannon, Simpson, Chao1, ACE, rarefaction), beta diversity (PCoA, NMDS, UPGMA), LEfSe (LDA > 4 for differential taxa)*	• α-diversity: no significant between OC vs. RJ group Phylum • *Firmicutes: Bacteroidota ratio*: ↑ in RJ group • *Actinobacteria*: ↓ in RJ group Family • *Muribaculacea*e: ↓ in RJ group • *Erysipelotrichaceae*: ↓ in RJ group Genus • *Bacteroides*: ↑ in RJ group • *Bifidobacterium*: ↓ in OC group; ↑ in RJ group • *Alistipes*: ↑ in RJ group • *Candidatus_Saccharimonas*: ↑ in RJ group • *Lactobacillus*: ↑ in RJ group • *Dubosiella:* ↓ in OC group; ↑ in RJ group

Abbreviations: N/A, not available; HCD, high cholesterol diet; IL-1β, Interleukin- 1 beta; IL-18, Interleukin-18; IL-5, Interleukin-5; IL-10, Interleukin −10; Bax, Bcl-2-associated X protein; Bcl-2, B-cell lymphoma 2; p-AKT, phosphorylated protein kinase B (Akt); Sirt1, Sirtuin 1; TLR4, toll-like receptor 4; MyD88, Myeloid differentiation primary response 88; p65, phosphorylated p65 subunit of NF-κB; IL-6, interleukin-6; TNF-α, tumor necrosis factor alpha; GOS, galacto-oligosaccharides; IL-17, interleukin-17; IP-10, Interferon gamma-induced protein 10; IL-13, interleukin-13; SOD, superoxide dismutase; MDA, malondialdehyde; ZYD, zengye decoction; p38 MAPK, p38 Mitogen-Activated Protein Kinase; JNK1, c-Jun N-terminal kinase 1; Nrf2, nuclear factor erythroid 2-related factor 2; GSH-Px, glutathione peroxidase; γ-H2AX, phosphorylated histone H2AX; P-ATM, phosphorylated Ataxia Telangiectasia Mutated kinase; SIRT6, sirtuin 6; Caspase-3, Cysteine-aspartic protease 3; Iba-1, Ionized calcium-binding adaptor molecule 1; GFAP, glial fibrillary acidic protein; NLRP3, NOD-like receptor family, pyrin domain containing 3; MCP-1, monocyte chemoattractant protein-1; COX-2, Cyclooxygenase-2; CAT, catalase.

### 3.4 Ageing parameters

In evaluating the interplay between natural products, ageing, and gut microbiota, researchers consistently focused on a set of biological parameters that reflect hallmark processes of ageing. Inflammatory markers were the most extensively measured secondary outcomes, evaluated in 82.4% of the literature (14 of 17 studies). These assessments heavily relied on cytokines such as interleukin-1β (IL-1β), interleukin-6 (IL-6), tumor necrosis factor-alpha (TNF-α), and interleukin-10 (IL-1), as well as signalling molecules like cyclooxygenase-2 (COX-2) and toll-like receptor 4 (TLR4), serving as key indicators of immunological changes linked to gut dysbiosis.

Parallel to this, oxidative stress markers were assessed in 35.3% of the studies (6 of 17) to capture shifts in redox homeostasis, frequently utilizing superoxide dismutase (SOD), catalase (CAT), glutathione peroxidase (GSH-Px), and malondialdehyde (MDA). In addition, 29.4% of the studies (5 of 17) incorporated behavioral and cognitive tests, including grip strength, Barnes maze, Y-maze, novel object recognition, and passive avoidance tasks to connect microbiota modulation with functional outcomes such as memory, learning, and motor performance. Interestingly, despite its central role in genomic stability, distinct markers of DNA damage such as phosphorylated H2A histone family member X (γ-H2AX), phosphorylated Ataxia Telangiectasia mutated kinase (p-ATM), were not explicitly assessed in any of the included studies, highlighting a major gap in the mechanistic framework. These findings are summarized in Table 5.

**TABLE 5:** Ageing parameters.

Author (year)	Oxidative stress markers	Inflammatory markers	DNA damage	Behavioral tests/Cognitive function tests
Crowe et al. (2020)	N/A	• Anti-phosphorylcholine antibodies ↓ in ES-62 + HCD-fed mice • Visceral fat eosinophils ↓ in ES-62 + HCD-fed • IL-1β (Type-1): ↑ chow + HCD-fed mice; ↓ with ES-62 + HCD-fed • IL-18 (Type-1): ↑ chow + HCD-fed mice; ↓ with ES-62 + HCD-fed • IL-5 (Type-2): ↑ chow + HCD-fed mice; ↓ with ES-62 + HCD-fed • IL-10 (Type-2): ↓ chow + HCD-fed mice; ↑ with ES-62 + HCD-fed	N/A	• Grip strength ↓ with age No effect with ES-62
Fang et al. (2021)	N/A	• Hippocampal neurons (NeuN staining): ↓ in ageing model (M) group; ↑ in probiotic combination (L & H) groups • Bax (pro-apoptotic): ↑ in M group; ↓ in L & H groups • Bcl-2 (anti-apoptotic): ↓ in M group; ↑ in L & H groups • p-AKT: ↓ in M group; ↑ in L & H groups • Sirt1: ↓ in M group; ↑ in L & H groups • TLR4 (hippocampus): ↑ in M group; ↓ in L & H groups • TLR4 (colon): ↑ in M group; ↓ in L & H groups • MyD88 (hippocampus): ↑ in M group; ↓ in L & H groups • MyD88 (colon): ↑ in M group; ↓ in L & H groups • p-p65/p65 (hippocampus): ↑ in M group; ↓ in L & H groups • p-p65 (colon): ↑ in M group; ↓ in L & H groups • IL-1β (hippocampus): ↑ in M group; ↓ in L & H groups • IL-6 (hippocampus): ↑ in M group; ↓ in L & H groups • TNF-α: ↑ in M group; ↓ in L & H groups	N/A	• Barnes Maze – Escape latency (s): slower learning/memory in ageing model (M) group; improved spatial learning and memory in probiotic combination (low and high) groups • Pole Test – Time to descend (s): ↑ time in M group; ↓ time in L&H groups • Open field – Total movement distance (cm): ↓ in M group; ↑ in control and L & H groups • Open field – Entries into central area (n): ↓ in M group; ↑ in control and L & H groups • Open field – Centra movement distance (cm): ↓ in M group; ↑ in control and L & H groups
Gao et al. (2023)	N/A	• IL-1β: ↑ in aged mice; ↓ in aged mice + WHH1889 • IL-6: ↑ in aged mice; ↓ in aged mice + WHH1889 • TNF-α: ↑ in aged mice; ↓ in aged mice + WHH1889	N/A	• Novel object recognition test (NORT) – recognition index (RI): ↓ in aged mice; ↑ in aged + WHH1889 mice • NORT – Discrimination index (DI): ↓ in aged mice; ↑ in aged + WHH1889 mice • Active Shuttle avoidance test (ASAT) – active escape times: ↓ from day 1–4 in aged mice; ↑ on day 2–4 in aged + WHH1889 mice • Y-Maze – total arm entries: ↓ in aged mice; ↑ in aged + WHH1889 mice • Y-Maze – spontaneous alternations (%): ↓ in aged mice; ↑ in aged + WHH1889 mice • Passive avoidance test (PAT) – Latency time: ↓ from day 1–3 in aged mice; ↑ from day 1–3 in aged + WHH1889 mice
He et al. (2019)	In liver, kidney, brain and serum • T-AOC: ↓ in AC & DG; ↑ MRPs • CAT: ↓ in AC & DG; ↑ MRPs • GSH-Px: ↓ in AC & DG; ↑ MRPs • SOD: ↓ in AC & DG; ↑ MRPs • MDA: ↑ in AC & DG; ↓ MRPs	In liver and kidney tissue • IL-1β: ↑ in AC & DG; ↓ MRPs group • IL-6: ↑ in AC & DG; ↓ MRPs group • TNF-α: ↑ in AC & DG; ↓ MRPs group	N/A	N/A
Arnold et al. (2021)	N/A	Overall effect of antibiotics • ↑ IL-17 (both diets, both ages) • ↑ IL-6 (both diets, both ages) Within antibiotic-treated groups • GOS diet: ↑ IL-6 (regardless of age) • Control diet: ↑ IL-6 in old vs. young mice IP-10 and Eotaxin • ↑ IP-10 & Eotaxin in Control diet (with antibiotics) • ↓ IP-10 & Eotaxin in GOS diet (with antibiotics) Age-related differences • ↑ IL-13 in young mice (regardless diet/antibiotics) • ↑ TNF-α gene expression in distal colon of old mice (control diet) • ↓ TNF-α gene expression in distal colon of old mice (with GOS diet)	N/A	N/A
Liu et al. (2019)	• ↑ SOD; ↓ MDA with ZYD vs. recovery No significant changes with ZYD vs. control	N/A	N/A	N/A
Ren et al. (2022)	N/A	• IL-10 ↑ in APF, APS, APRS (APRS > APS > APF) • TNF-α ↓ in APF, APS, APRS (APRS > APS > APF) • IL-6 ↓ in APF, APS, APRS • IL-1β ↓ in APF, APS, APRS • p38 MAPK ↓ in APF, APS, APRS • JNK1 ↓ in APF, APS, APRS	N/A	N/A
Shenghua et al. (2020)	N/A	• IL-6: ↑ in ageing; ↓ in FTZ group • TNF-α: ↑ in ageing; ↓ in FTZ group • Cyclooxygenease-2 (COX-2): ↑ in ageing; ↓ in FTZ group • Activatorprotein-1 (AP-1): ↑ in ageing; ↓ in FTZ group	N/A	N/A
Xiang et al. (2023)	N/A	N/A	N/A	N/A
Xie et al. (2020)	• Nrf2: ↓ in model and control group; ↑ in H-SCML & M-SCML • GSH-Px: ↓ in model and control group; ↑ all SCML groups • SOD-1: ↓ in model and control group; ↑ in H-SCML & M-SCML	N/A	N/A	N/A
Yang et al. (2020)	N/A	Hippocampus: • *Ibα-1* protein: ↑ in AGE group • GFAP protein: ↓ in Q group and AQ group Cortex: • GFAP: ↑ in AGE vs. CON; ↓ in AQ vs. AGE NLRp3: no significant differences	N/A	• Escape latency (navigation trials): no significant differences • Spatial probe trial: ↓ time in target quadrant in AGE group; ↑ time in quercetin-treated (AQ) • Swimming route observation: aimless swimming in AGE group; direct swimming in AQ group
Yu et al. (2023)	N/A	• TNF-α: ↓ with L. plantarum FLPL05; ↑ with L. plantarum FLPL0302 • IL-6: no significant change with L. plantarum FLPL05; ↑ with L. plantarum FLPL0302 No evidence of inflammation alleviation in aged mice	N/A	N/A
Zhang et al. (2021a)	• MDA: ↑ in SD, WS & FMT vs. CON; ↓ in WS vs. SD • SOD: ↓ in SD, WS & FMT vs. CON; ↑ in WS vs. SD • GSH-Px: ↓ in SD, WS & FMT vs. CON	• IL-2: ↓ in SD & WS vs. CON; ↑ in WS vs. SD • IL-4: no significance • IL-6: ↑ in SD, WS & FMT vs. CON; ↓ in WS vs. SD • IL-10: ↑ in SD, WS & FMT vs. CON • TNF-α: ↑ in SD, WS & FMT vs. CON; ↓ in WS vs. SD • INF-γ: no significance	N/A	• Avoidance response: Con & WS groups ↑ avoidance ratio from day 4 training • Passive avoidance retention: SD group ↑ retention vs. WS and FMT from day 3 • WS & FMT groups have no significant difference vs. Con group
Zhang et al. (2024)	• SOD: ↓ in D-gal group; ↑ in M + A & M + H • GSH-Px: ↓ in D-gal group; ↑ in M + A & M + H • Total antioxidant capacity (T-AOC): ↓ in D-gal group; ↑ in M + A & M + H • MDA: ↑ in D-gal group; ↓ in M + A, M + H & M + HR	• TNF-α: ↑ in D-gal group; ↓ in M + A, M + H & M + HR • IL-6: ↑ in D-gal group; ↓ in M + A, M + H & M + HR • IL-1β: ↓ in D-gal group; ↑ in M + HR	N/A	N/A
Chen et al. (2024a)	N/A	• MCP-1: ↑ in aged ob/ob; ↓ after TSF • COX-2: ↑ in aged ob/ob; ↓ after TSF • IL-6: ↑ in aged ob/ob; ↓ after TSF • TNF-α: ↑ in aged ob/ob; ↓ after TSF	N/A	N/A
Gao et al. (2024)	• MDA: ↑ in SAMP8-WT; ↑ with Rg2 treatment • T-SOD: ↓ in SAMP8-WT, ↑ with Rg2 treatment • CAT: ↓ in SAMP8-WT, ↑ with Rg2 treatment	• TNF-α: ↑ in SAMP8-WT; ↓ with Rg2 treatment • IL-1β: ↑ in SAMP8-WT; ↓ with Rg2 treatment • IL-18: ↑ in SAMP8-WT; ↓ with Rg2 treatment	N/A	N/A
Chen et al. (2024b)	N/A	• ↓ TNF-α with RJ • ↓ IL-6 with RJ • ↑ IL-10 with RJ	N/A	N/A

Abbreviations: N/A, not available; HCD, high cholesterol diet; IL-1β, Interleukin- 1 beta; IL-18, Interleukin-18; IL-5, Interleukin-5; IL-10, Interleukin −10; Bax, Bcl-2-associated X protein; Bcl-2, B-cell lymphoma 2; p-AKT, phosphorylated protein kinase B (Akt); Sirt1, Sirtuin 1; TLR4, toll-like receptor 4; MyD88, Myeloid differentiation primary response 88; p65, phosphorylated p65 subunit of NF-κB; IL-6, interleukin-6; TNF-α, tumor necrosis factor alpha; GOS, galacto-oligosaccharides; IL-17, interleukin-17; IP-10, Interferon gamma-induced protein 10; IL-13, interleukin-13; SOD, superoxide dismutase; MDA, malondialdehyde; ZYD, zengye decoction; p38 MAPK, p38 Mitogen-Activated Protein Kinase; JNK1, c-Jun N-terminal kinase 1; Nrf2, nuclear factor erythroid 2-related factor 2; GSH-Px, glutathione peroxidase; γ-H2AX, phosphorylated histone H2AX; P-ATM, phosphorylated Ataxia Telangiectasia Mutated kinase; SIRT6, sirtuin 6; Caspase-3, Cysteine-aspartic protease 3; Iba-1, Ionized calcium-binding adaptor molecule 1; GFAP, glial fibrillary acidic protein; NLRP3, NOD-like receptor family, pyrin domain containing 3; MCP-1, monocyte chemoattractant protein-1; COX-2, Cyclooxygenase-2; CAT, catalase.

A comparison across the experimental designs reveals distinct, model-specific patterns in outcome assessment that reflect the biological strengths of each model. First, oxidative stress parameters were predominantly prioritized in chemically induced D-galactose models, with 80% of these studies (four of the five) assessing redox markers such as SOD and MDA. In contrast, exclusively naturally aged models focused heavily on immunological shifts; seven out of seven studies reported inflammatory outcomes, while none assessed oxidative stress. Notably, a multi-parametric overlap was observed in genetically accelerated models, which frequently bridged the redox-immune axis by assessing both oxidative stress and chronic inflammation simultaneously. Finally, behavioral and cognitive assessments were disproportionately concentrated in genetically accelerated and diet-induced ageing models, reflecting their specific suitability for manifesting rapid, quantifiable functional declines. Ultimately, these findings highlight how the choice of ageing model inherently dictates the biological endpoints investigated.

### 3.5 Assessment of the risk of bias (RoB) tool for animal studies

The methodological quality of the 17 included studies was evaluated using the SYRCLE Risk of Bias (ROB) tool, which assesses bias across 10 specific domains (Table 6). A domain-wise analysis reveals specific methodological strengths and critical weaknesses across the evaluated literature. According to Figure 1, all records (100%) demonstrated a low risk of bias in allocation concealment (question 3), attrition bias (question 8), reporting bias (question 9), and other biases (question 10). A low risk of bias was also frequently observed for random housing (question 4; ∼95%), sequence generation (question 1; ∼80%), and baseline characteristics (question 2; ∼55%).

**TABLE 6:** Risk assessment using SYRCLE’s tool of risk of bias assessment.

Authors (Year)	1	2	3	4	5	6	7	8	9	10
Crowe et al. (2020)	Y	Y	Y	Y	N/A	N/A	N/A	Y	Y	Y
Fang et al. (2021)	N/A	N	Y	Y	N/A	N/A	N/A	Y	Y	Y
Gao et al. (2023)	N/A	N	Y	Y	N/A	N/A	N/A	Y	Y	Y
He et al. (2019)	Y	Y	Y	Y	N/A	N/A	N/A	Y	Y	Y
Arnold et al. (2021)	Y	N/A	Y	Y	N/A	N/A	N/A	Y	Y	Y
Liu et al. (2019)	Y	Y	Y	Y	N/A	N/A	N/A	Y	Y	Y
Ren et al. (2022)	Y	Y	Y	Y	N/A	N/A	N/A	Y	Y	Y
SShenghua et al. (2020)	Y	Y	Y	Y	N/A	N/A	N/A	Y	Y	Y
Xiang et al. (2023)	Y	Y	Y	Y	N/A	N/A	N/A	Y	Y	Y
Xie et al. (2020)	Y	N	Y	Y	N/A	N/A	N/A	Y	Y	Y
Yang et al. (2020)	Y	Y	Y	Y	N/A	N/A	N/A	Y	Y	Y
Yu et al. (2023)	Y	Y	Y	Y	N/A	N/A	N/A	Y	Y	Y
Zhang et al. (2021a)	N/A	Y	Y	Y	N/A	N/A	N/A	Y	Y	Y
Zhang et al. (2024)	Y	N	Y	Y	N/A	N/A	N/A	Y	Y	Y
Chen et al. (2024a)	Y	N	Y	Y	N/A	N/A	N/A	Y	Y	Y
Gao et al. (2024)	Y	Y	Y	Y	N/A	N/A	N/A	Y	Y	Y
Chen et al. (2024b)	Y	Y	Y	Y	N/A	N/A	N/A	Y	Y	Y

Abbreviations: Symbol “Y” with green-coloured box indicates compliance, “N” with red-coloured boxes indicates non-compliance, and “N/A” with yellow-coloured boxes indicate that data were unavailable.

However, a severe domain-level weakness was identified regarding performance and detection bias. Information concerning the blinding of investigators (question 5), randomization of outcome assessment (question 6), and blinding of outcome assessors (question 7) was consistently unavailable (‘N/A’) across all 17 studies. This systemic lack of reporting on blinding and randomization significantly increases the overall risk of bias, highlighting a major methodological limitation in the current preclinical literature.

## 4 Discussion

This review explored how natural product interventions influence gut microbiota in ageing animal models, offering valuable insight into the role of dietary and, botanical drugs in alleviating age-related dysbiosis. The findings indicate that such interventions can positively affect microbial composition, diversity, and function, thereby reducing oxidative stress and inflammation while improving cognitive behavioral outcomes.

### 4.1 Natural products restore microbial balance in ageing

A recurring observation across the studies is that natural products can significantly alter gut microbiota composition. Interventions with natural products such as polyphenols (Yang et al., 2020; Zhang et al., 2024; Xiang et al., 2023), flavonoids (Gao et al., 2024), probiotics (Gao et al., 2023; Yu et al., 2023; Fang et al., 2021), prebiotics (Ren et al., 2022; Arnold et al., 2021), and traditional Chinese medicine (Chen D. Q. et al., 2024; Shenghua et al., 2020; Gao et al., 2024; Liu et al., 2019) were shown to suppress harmful bacteria such as *Helicobacter* and *Desulfovibrio*, while promoting beneficial groups, including *Lactobacillus, Bifidobacterium,* and *Akkermansia*. While several studies highlighted as restored Firmicutes/Bacteroidetes (F/B) ratio, it is increasingly recognized that relying on such broad phylum-level metrics is an oversimplification of complex microbial dynamics. Contemporary microbiome research emphasizes that the F/B ratio provides limited functional insight, underscoring the need for higher-resolution taxonomic profiling rather than reductionist phylum-level ratios. Notably, formulations containing multiple probiotic strains or botanical drugs produced a wider range of effects than single-strain or isolated compounds, suggesting potential synergistic benefits from multi-component approaches.

Ageing is a multifaceted biological process that profoundly influences gut microbiota composition and function. With advancing age, the gut microbial community exhibits marked reductions in diversity, along with shifts in the relative abundance of specific bacterial taxa. According to Li et al., ageing-associated dysbiosis is accompanied by diminished production of short-chain fatty acids (SCFAs), metabolites crucial for sustaining gut health and physiological balance (Li et al., 2024).

A healthy gut microbiome is characterized by a diverse and well-balanced microbial community that contributes to maintaining host physiological functions (Kyrochristou et al., 2026). In contrast, gut dysbiosis is typically defined by reduced microbial diversity, diminished abundance of beneficial taxa, and/or an increased prevalence of potentially pathogenic microorganisms (Shen et al., 2020). The human gut microbiota is primarily dominated by the phyla *Firmicutes* and *Bacteroidetes*, which together account for over 90% of the microbial community, with additional contributions from subdominant phyla such as *Proteobacteria, Actinobacteria,* and *Verrucomicrobia* (Qin et al., 2010). The gut microbial community exhibits a high degree of resilience, with transient disturbances often followed by a swift restoration of its baseline composition (Candela et al., 2012). Disruptions in the gut microbiota linked to ageing or disease can compromise nutrient absorption and alter key microbial functions essential to host health. These compositional shifts not only impair metabolic and immune processes but may also contribute to progressive functional decline, thereby increasing susceptibility to a range of age-associated pathologies. These compositional shifts not only impair metabolic and immune processes but may also contribute to progressive functional decline, thereby increasing susceptibility to a range of age-associated pathologies (Finlay et al., 2019).

### 4.2 Links between microbiota modulation and ageing parameters

The oxidative-inflammatory theory of ageing (De la Fuente and Miquel, 2009) proposes that the age-related rise in oxidative stress (Harman, 1956; Barja, 2002) is closely connected to chronic low-grade inflammation, commonly termed “inflammaging” (Franceschi et al., 2000), through interactions with the immune system. Elevated oxidative stress with ageing is known to impair normal cellular function. Since oxidation and inflammation are interdependent processes, heightened oxidative stress in immune cells promotes the release of proinflammatory mediators, ultimately leading to a persistent state of oxidative and inflammatory stress in ageing (De la Fuente and Miquel, 2009). This cycle is further aggravated by dysbiosis, forming a feedback loop in which oxidative conditions drive further microbial imbalance, thereby perpetuating oxidative stress and inflammation (Pan et al., 2022; Li et al., 2023).

Building upon these foundational theories of systemic ageing, emerging evidence explicitly links this oxidative-inflammatory cycle directly to the gut ecosystem. Gut microbiota dysbiosis is now recognized to actively contribute to oxidative stress by increasing ROS production, disrupting gut barrier integrity, and promoting systemic inflammation (Семенова et al., 2024; Luca et al., 2019; Dumitrescu et al., 2018). Conversely, beneficial microbial metabolites, such as short-chain fatty acids (SCFAs), can help maintain redox balance and mitigate oxidative damage (McBeth et al., 2025). As these effects extend beyond the gastrointestinal tract, modulation of the gut microbiota may also influence cognitive function and age-related neurodegenerative processes (McBeth et al., 2025). As these effects extend beyond the gastrointestinal tract, modulation of the gut microbiota may also influence cognitive function and age-related neurodegenerative processes (Dumitrescu et al., 2018; Maffei et al., 2017).

This review highlights the therapeutic potential of targeting microbiota. Natural product supplementation was frequently linked to improvements in parameters associated with ageing. Several studies reported enhanced antioxidant defenses, with increased activities of SOD (Xie et al., 2020; Gao et al., 2024; Liu et al., 2019; Zhang et al., 2024; He et al., 2019), CAT (Gao et al., 2024; He et al., 2019), and GSH-Px (Xie et al., 2020; Zhang et al., 2024; He et al., 2019), accompanied by reduced levels of the lipid peroxidation marker MDA (Liu et al., 2019; Zhang et al., 2024; He et al., 2019). These findings are in line with previous studies showing that gut microbiota dysbiosis contributes to oxidative stress, cognitive decline, vascular dysfunction, and metabolic disturbances during ageing (Chen et al., 2022; Brunt et al., 2019; Jin et al., 2025; Liu et al., 2022). Conversely, interventions such as polysaccharides, probiotics, and polyphenols have been reported to restore microbial balance and alleviate oxidative stress, highlighting the therapeutic potential of targeting the gut microbiota (Pan et al., 2022; Zhou et al., 2021; Conley et al., 2016).

Alongside oxidative stress, inflammation is a key hallmark of gut microbiota-ageing interactions. Dysbiosis has been shown to promote elevated production of pro-inflammatory cytokines, including IL-6 and TNF-α, thereby driving the development of chronic inflammation (Odamaki et al., 2016; Fransen et al., 2017). Beyond oxidative stress, natural product supplementation also exerted anti-inflammatory effects. Pro-inflammatory cytokines such as TNF-α and IL-6 were consistently reduced (Zhang J. et al., 2021; Chen D. Q. et al., 2024; Shenghua et al., 2020; Gao et al., 2024; Gao et al., 2023; Ren et al., 2022; Zhang et al., 2024; He et al., 2019; Chen L. et al., 2024; Yu et al., 2023; Fang et al., 2021), while the levels of the anti-inflammatory cytokine IL-10 were elevated (Crowe et al., 2020; Zhang J. et al., 2021; Ren et al., 2022; Chen L. et al., 2024), highlighting the role of gut-immune interactions in shaping systemic inflammation. Specific microbial shifts were linked to pro-inflammatory markers, as shown by Conley et al., who reported correlations between taxa and serum MCP-1 in ageing mice (Conley et al., 2016). Similarly, Kim et al. Specific microbial shifts were linked to pro-inflammatory markers, as shown by Conley et al., who reported correlations between taxa and serum MCP-1 in ageing mice (Conley et al., 2016). Similarly, Kim et al. showed that age-related microbial changes can accelerate “inflammaging” through *Firmicutes*-derived lipopolysaccharides (LPS), which activate Toll-like receptor 4 (TLR4) signalling and elevate pro-inflammatory cytokines such as TNF-α (Kim et al., 2016). More recently, a study identified microbial markers of inflammation, including a negative association between the abundance of *Oscillospira* and MCP-1, suggesting that enrichment of beneficial taxa could help counteract inflammatory states (Dou et al., 2025). Dietary influence also plays a role, as reviewed by a previous study, which noted that while specific dietary interventions improved gut microbiota diversity, the effects on systemic inflammatory markers were inconsistent, underscoring the complexity of diet-microbiota-immune interactions (Telle‐Hansen et al., 2018).

Behavioural assessments in animal models are essential for exploring ageing-related processes and providing valuable insights into both cognitive and physical changes over time. This review showed that natural product interventions improved outcomes in behavioural tests, including the Barnes maze (Fang et al., 2021), Y-maze (Gao et al., 2023), and novel object recognition (Gao et al., 2023; Fang et al., 2021). These findings emphasize the relevance of the gut-brain axis in protecting against age-associated cognitive decline. Evidence from studies such as Zhu et al., indicates that faecal microbiota transplantation can reduce frailty and even reverse cognitive impairments in ageing mice, highlighting a direct link between gut microbial health and cognitive performance (Zhu et al., 2024). Moreover, a strong connection exists between gut microbiota, inflammationinflammation, and behavioural outcomes in ageing. A previous study by Fransen et al., demonstrated that alterations in gut microbiota composition in older individuals contribute to systemic inflammation, which in turn can compromise both cognitive and physical functions (Fransen et al., 2017). The persistent inflammatory state commonly associated with ageing negatively affects both cognitive function and motor performance, as demonstrated in behavioural assessments like grip strength tests (Yokozuka et al., 2022). This test is crucial, since grip strength is widely used as an indicator of overall physical capacity and general health status in older adults (Yokozuka et al., 2022).

The results highlight how gut microbiota links oxidative stress, inflammation, and behavioural decline during ageing. Interventions that target the microbiota through probiotics, natural products, or diet offer a potential means to rebalance redox status, ease inflammaging, and support healthier ageing.

### 4.3 Study variabilities

A critical challenge in synthesizing the current evidence is the high degree of methodological heterogeneity across experimental approaches. Among the various sources of heterogeneity, the choice of animal model most strongly affects the interpretation of outcome and their subsequent translational potential. Naturally aged models, chemically induced models (D-galactose), and genetically accelerated models (SAMP8, ob/ob) exhibit fundamentally different baseline microbiota profiles and physiological trajectories. Accelerated models rapidly manifest acute oxidative stress and metabolic disturbances, whereas naturally aged rodents undergo a gradual, cumulative inflammatory decline. Because the baseline physiological and microbial “starting points” differ so drastically, the modulatory effects of a natural product in a D-galactose model cannot be reliably equated to its effects in a natural aged model.

Furthermore, while accelerated and induced models are valuable for time-efficient, mechanistic probing, they carry significant translational limitations. Their artificially induced pathways frequently diverge from the complex, multifactorial progression of natural human ageing. Consequently, a natural product that successfully reverses acute, chemically induced dysbiosis in a matter of weeks may not exhibit the same therapeutic efficacy when applied to the lifelong, gradual physiological decline seen in older adults. Therefore, while accelerated models serve as useful complementary tools, natural ageing models remain the most translationally relevant framework for predicting human clinical outcome.

A major source of variability lies in the methodological approaches to microbiota profiling. Most studies used 16S rRNA sequencing, though targeting different regions (V3-V4, or full-length) and sequencing platforms (Illumina HiSeq/MiSeq, PacBio SequenII). Some employed shotgun metagenomics for greater resolution, while functional predictions were carried out in selected studies using tools such as PICRUSt and STAMP. The choice of bioinformatics pipelines (QIIME, QIIME2, MG-RAST, LEfSe) further added to methodological heterogeneity. However, the implications of this methodological divergence extend beyond mere technical differences; they introduce systematic biases that severely restrict cross-study comparability. Specifically, targeting different 16S hypervariable regions (such as V3-V4 versus full-length) can result in the preferential amplification or underrepresentation of specific bacterial taxa, inherently skewing relative abundance profiles. Furthermore, employing distinct bioinformatics, pipelines, and reference databases leads to variations in taxonomic annotation, meaning that identical raw sequences could be classified differently depending on the study. Consequently, absolute quantitative comparisons of microbial shifts, even at higher taxonomic levels, cannot be reliability equated across the included studies.

Accurate assessment of the gut microbiome is fundamental to understanding its role in ageing and health. Faecal sampling remains the most common approach due to its convenience and ability to capture broad microbial profiles, though it may not fully reflect regional gut communities (Salosensaari et al., 2021). To address this limitation, multi-site sampling of regions such as the cecum and colon has been used to provide more comprehensive insights into microbial diversity, while functional predictions were carried out in selected studies using tools such as PICRUSt and STAMP (Xiang et al., 2023).

Finally, heterogeneity in outcome selection limits comparability. While oxidative stress was a consistent focus, the markers reported were diverse. Enzymatic antioxidants such as SOD, CAT, and GSH-Px were frequently measured (Xie et al., 2020; Liu et al., 2019; Zhang et al., 2024; He et al., 2019) often alongside MDA as a marker of lipid peroxidation. Some studies extended analysis to specific redox-related metabolites or microbial pathways predicted to influence oxidative stress (Jin et al., 2025). Evaluating oxidative stress parameters provides critical insights into the interaction between gut microbiota and host health during ageing. MDA remains a reliable indicator of lipid peroxidation, while elevated levels reflect oxidative damage and compromised microbial balance (Gao et al., 2018). Antioxidant enzymes such as SOD, GSH-Px, and catalase serve as markers of the host’s defence capacity, and their modulation has been linked to shifts in microbial composition (Morshedi et al., 2020). In parallel, while short-chain fatty acids (SCFAs), particularly butyrate, play a protective role by maintaining epithelial integrity and mitigating oxidative stress, it is imperative to recognize that their physiological effects are highly context-dependent. Rather than acting as uniformly beneficial metabolites, the impact of SCFAs relied heavily on their relative concentrations, the specific tissue microenvironment, and the baseline inflammatory status of the host. Generalizing SCFAs as universally therapeutic oversimplifies the complex, bidirectional nature of host-microbe interactions during ageing (Tsao et al., 2021).

Alongside oxidative stress, the evaluation of inflammation also featured diverse biomarker panels. Inflammation was typically assessed through pro-inflammatory cytokines (e.g., TNF-α and IL-6), anti-inflammatory cytokines (e.g., IL-10), chemokines such as MCP-1, and signaling pathways including TLR4 and NF-κB. While these diverse parameters form a robust framework for evaluating gut-immune interactions, the lack of a universally standardized inflammatory panel across the studies further complicates direct comparisons of therapeutic efficacy. Ultimately, the multifaceted heterogeneity, spanning animal models, sequencing platforms, and outcome selection, highlights a critical need for standardized preclinical frameworks to enable rigorous, comparative meta-analyses in future research. Across the included studies, natural product interventions generally reduced pro-inflammatory markers while enhancing anti-inflammatory responses, suggesting a beneficial role in mitigating age-related inflammation associated with gut dysbiosis. Collectively, these findings support the interplay between gut microbiota modulation, oxidative stress, and inflammatory processes in ageing.

### 4.4 Mechanistic insights

Evidence from the included studies suggests that natural product interventions may alleviate ageing phenotypes through modulation of oxidative stress, inflammation, and gut microbiota composition. At the host-microbe interface, natural products consistently improved gut microbial composition and function. Interventions promoted beneficial taxa, including *Akkermansia* and SCFA-producing bacteria, while reducing the abundance of potentially pathogenic microorganisms (Arnold et al., 2021; Zhao et al., 2017; Collado et al., 2007). This restoration of microbial diversity is critical for enhancing intestinal barrier integrity, which helps prevent the systemic translocation of endotoxins (such as lipopolysaccharides) that trigger immune activation.

Beyond the modulation of specific taxonomic groups, the therapeutic efficacy of natural products must also be viewed through the broader lens of ecological stability and microbiota resilience. A healthy gut microbiome is characterized by a high degree of ecological resilience, relying on complex, highly connected co-occurrence networks that enable a swift restoration of its baseline composition following transient environmental disturbances (Fassarella et al., 2021). However, the ageing process inherently disrupts this equilibrium, leading to a fragmented microbial network that is highly vulnerable to physiological stressors (Chen L. et al., 2024). The synthesized evidence suggests that natural product interventions function as broad ecological modulators. By enriching keystone taxa and suppressing pathogenic overgrowth, these interventions help rebuild the complex, highly connected network topology of the youthful gut microbiome (Chen L. et al., 2024). This restored ecological resilience effectively buffers the intestinal environment against age-related decline, ensuring that the host maintains robust metabolic and immunological homeostasis.

By restoring barrier integrity, natural products interrupt the harmful feedback loop between redox imbalance and inflammaging (Untersmayr et al., 2022). Across diverse intervention types, improvements in antioxidant status were consistently accompanied by reductions in pro-inflammatory mediators, indicating that microbiota modulation directly contributes to the attenuation of systemic age-related oxidative and inflammatory processes. The benefits of reducing systemic inflammation and oxidative stress also extended to the gut-brain axis. Several studies reported improvements in cognitive outcomes and behavioral tests following natural product interventions. However, while these cognitive improvements are frequently hypothesized to operate via the microbiota-gut-brain axis, such claims extend beyond the current evidence base. Because the included studies relied primarily on phenotypic behavioral observations without comprehensive neurochemical profiling or microbiome-depletion models, the precise role of the gut-brain axis in mediating these neurological benefits remains correlative rather than definitively proven (Vatner et al., 2020; Ng et al., 2021; Rashid et al., 2013; Zhu et al., 2014).

Finally, while genomic stability is widely recognized as a key hallmark of ageing (López-Otín et al., 2013), none of the included studies explicitly evaluated DNA damage or DNA repair markers. Consequently, the direct relationship between natural product interventions, gut microbiota modulation, and genomic stability remains unclear and warrants further investigation. Collectively, these findings suggest that natural products mitigate age-related decline through coordinated microbiota-mediated, antioxidant, and anti-inflammatory mechanisms, while their definitive roles in gut-brain axis signaling and genomic stability require far more rigorous experimental validation.

Furthermore, a major mechanistic gap across the synthesized literature is the widespread failure to distinguish direct pharmacological effects from true microbiota-mediated mechanisms. Because many natural products contain highly bioavailable compounds that enter systemic circulation, the observed anti-ageing benefits such as the attenuation of systemic oxidative stress or inflammation, may occur directly at the tissue levels, independent of the gut microbiota. To definitively establish causality and prove that the altered microbial ecosystem is actively driving the physiological improvements, experimental designs must utilize germ-free animal models, antibiotic-induced microbiota depletion, or fecal microbiota transplantation (FMT). However, these rigorous causality-driven models are largely absent from the current evidence base. Among the included studies, only two notable exceptions attempted to isolate the microbiome’s role. Zhang et al. utilized an FMT model, demonstrating that the transfer of feces from mice fed a functional food (*Wushen*) successfully mimicked the intestinal microbiota status of the treated mice and transferred corresponding immunological benefits to the recipients (Zhang J. et al., 2021). Similarly, Arnold et al. utilized an antibiotic depletion model, proving that the host’s inflammatory status (such as serum IL-6 and IL-17 levels) was profoundly dependent on the microbial community, with antibiotics having a more significant impact on systemic inflammation than the prebiotic diet itself (Arnold et al., 2021). Aside from these isolated examples, none of the 17 included studies employed germ-free ageing models. Consequently, while the concurrent shifts in gut microbiota and host ageing biomarkers are strongly correlated, the assumption that these natural products operate primarily through microbiota-mediated pathways remains largely inferential and requires substantial future validation using germ-free paradigms.

### 4.5 Methodological quality

The RoB assessment for animal studies included in this systematic review was performed using SYRCLE’s tool. This assessment was done individually by two authors to ensure consistency and prevent any biases during the assessment. Consistent with our domain-wise analysis, the most critical methodological gap identified was the universal lack of reporting regarding blinding and randomization during outcome assessment. None of the studies included in this review explicitly reported the practice of blinding investigators or outcome assessors. This omission is highly concerning, as the lack of blinding is widely known to artificially inflate reported therapeutic effects. Without it, subconscious observer bias can lead to the systemic overestimation of efficacy, particularly in subjective measurements such as behavioral assessments and manual histological scoring.

Furthermore, this lack of blinding is often compounded by the potential for selective outcome reporting. In the absence of blinded, pre-registered protocols, there is a substantial risk that research may preferentially publish statistically significant biomarker shifts while omitting null or negative findings, thereby skewing the overall evidence base to appear overwhelmingly positive. These findings reflect a persistent, low awareness and compliance rate regarding the ARRIVE guidelines among basic medical researchers. Strict adherence to the ARRIVE guidelines is critically warranted, not merely as an administrative checklist, but as a fundamental safeguard to ensure transparent reporting, mitigate domain-specific biases, and ultimately improve the reproducibility and translational reliability of preclinical animal studies (Hooijmans et al., 2014).

### 4.6 Methodological limitations and future directions

While the synthesized evidence highlights the potential of natural products to mitigate age-related dysbiosis, it is imperative to critically appraise the methodological limitations of the included studies. First, the majority of the studies relied on relatively small sample sizes, typically ranging from 6 to 24 animals per experimental group, which inherently reduces statistical power and increases the risk of overestimating therapeutic effect sizes. Second, as highlighted in our domain-wise risk of bias assessment, there is a systemic lack of reporting regarding the blinding of investigators and assessors, coupled with incomplete details on randomization procedures. This significantly increases the risk of performance bias and detection bias across the 17 studies. Third, interventions were predominantly short-term (often 4–12 weeks), particularly in chemically induced and genetically accelerated models, limiting our understanding of the long-term efficacy, safety, and durability of these natural products across the natural lifespan. Furthermore, the immense heterogeneity in animal models, dosages, and intervention types results in limited reproducibility and makes direct comparability across studies highly challenging. Additionally, A critical, widely underreported limitation across the preclinical microbiome literature is the failure to account for environmental confounders. Baseline microbiota composition is profoundly influenced by vendor-associated differences, while co-housing introduces significant “cage effects” due to rodent coprophagy. Because the included studies rarely reported statistical controls for these facility-driven variables, environmental variance may heavily confound the reported microbial shifts.

Mechanistically, the current preclinical landscape also suffers from an overreliance on 16S rRNA sequencing. While this technique is highly effective for broad taxonomic profiling, it typically restricts interpretation to the genus level. This limitation risks overgeneralizing microbial functions, as therapeutic efficacy and metabolic outputs are frequently highly strain specific. Furthermore, several included studies inferred functional and metabolic outcomes using computational prediction tools such as PICRUSt based solely on 16S taxonomic data. Because these predictive tools rely on established reference genomes, they cannot account for real-time host-microbe metabolic interactions or novel strain adaptations and therefore cannot replace direct empirical measurements. Consequently, the exact metabolic pathways induced by these natural products remain largely predictive rather tham empirically confirmed through direct metabolomics or shotgun metagenomics. Moreover, because 16S rRNA sequencing exclusively targets bacterial populations, the potential modulatory effects of natural products on the archaea, fungal (mycobiome), and viral (virome) communities remain entirely unassessed in the current literature, despite their recognized roles in maintaining gut equilibrium. Finally, as with many systematic reviews evaluating therapeutic interventions, there is a distinct risk of publication bias. Studies with null or negative findings regarding natural products are statistically less likely to be published, which may skew the available literature toward an overwhelmingly positive perspective. Furthermore, the exclusion of one article that was unable to be retrieved during our full-text screening process introduces a minor risk of retrieval bias. However, given the broad scope of the 17 included studies, the omission of a single record is unlikely to fundamentally alter the overall mechanistic consensus of this review.

Beyond these methodological limitations, translating these promising preclinical findings into practical clinical applications for human ageing faces several significant hurdles. While rodents are invaluable experimental models, substantial differences exist between rodent and human gastrointestinal anatomy, transit times, and basal microbiota composition, which inherently limit direct generalizability. Other than that, translating therapeutic efficacy requires rigorous allometric dose scaling; the relatively high concentrations of natural products frequently administered to rodents may not be pharmacologically feasible, bioavailable, or safe in humans. Moreover, unlike the highly controlled environments and standardized diets of animal facilities, the human gut microbiota is profoundly shaped by lifelong variability in diet, genetics, lifestyle, and polypharmacy. This high degree of environmental variance can easily mask or alter the modulatory effects of natural product interventions in real-world populations. Additionally, there are substantial regulatory and safety considerations associated with complex herbal therapies, such as Traditional Chinese Medicine formulations. Ensuring batch-to-batch consistency, standardizing the concentration of active bioactive compounds, and ruling out potential herb-drug interactions in older adults taking multiple medications remain major regulatory challenges. Finally, while probiotics demonstrate clear, consistent efficacy in inbred rodent models, reproducing these effects in elderly clinical populations is notoriously difficult due to extreme inter-individual differences in baseline microbiome profiles (resulting in ‘responder’ vs. ‘non-responder’ phenotypes) alongside age-related declines in immune function. Therefore, carefully designed, personalized clinical trials are essential to determine the true translational value and safety of these natural products for human ageing. Mechanistic investigations were also fragmented, often limited to inflammatory cytokines or oxidative stress markers, and lacked integration across biological systems. The relationship between microbiota remodeling and host pathways such as mitochondrial function, epigenetic regulation, and tryptophan-kynurenine metabolism remains poorly defined. Markers of genomic stability, including DNA damage and repair responses, were rarely explored, even though they are central to ageing biology. Similarly, functional outcomes such as cognition, memory, and motor performance were not consistently evaluated, leaving the gut-brain axis underrepresented in the current evidence base. It is also important to note that several plant-derived interventions included polyphenolic compounds, which may exhibit non-specific bioactivities in certain experimental settings. Therefore, mechanistic conclusions from *in vitro* findings should be interpreted with caution, particularly when pharmacological relevance has not been confirmed *in vivo*. Although this systematic review focused on animal studies evaluating physiological and gut microbiota-related outcomes, inconsistencies in phytochemical characterization and limited mechanistic validation across the included studies remain important limitations of the current evidence. Another important consideration is the limited reporting of safety and toxicological outcomes. Although the studies reported beneficial effects on gut microbiota composition and ageing-related parameters, the assessment of adverse effects, tolerability and long-term safety was lacking. This limits the ability to evaluate the translational relevance of these interventions, particularly for long-term use in the ageing population.

Future work should therefore prioritize longitudinal studies in naturally aged models to confirm sustained effects and to identify critical time windows for intervention. A broader use of multi-omics approaches integrating metagenomics, metabolomics, lipidomics, and transcriptomics would allow a more comprehensive mapping of host-microbe interactions. Inclusion of both sexes and genetically diverse strains will be necessary to capture variability in response, while dose-response and comparative studies could help clarify the relative efficacy of plant-, microbial-, animal-, and marine-derived products. In addition, future preclinical studies should incorporate safety and toxicological assessments alongside the efficacy evaluations. Ultimately, translation to humans will requirecarefully designed clinical studies in older adults to determine whether these preclinical benefits can be realized in practice.

## 5 Conclusion

**FIGURE 3:**
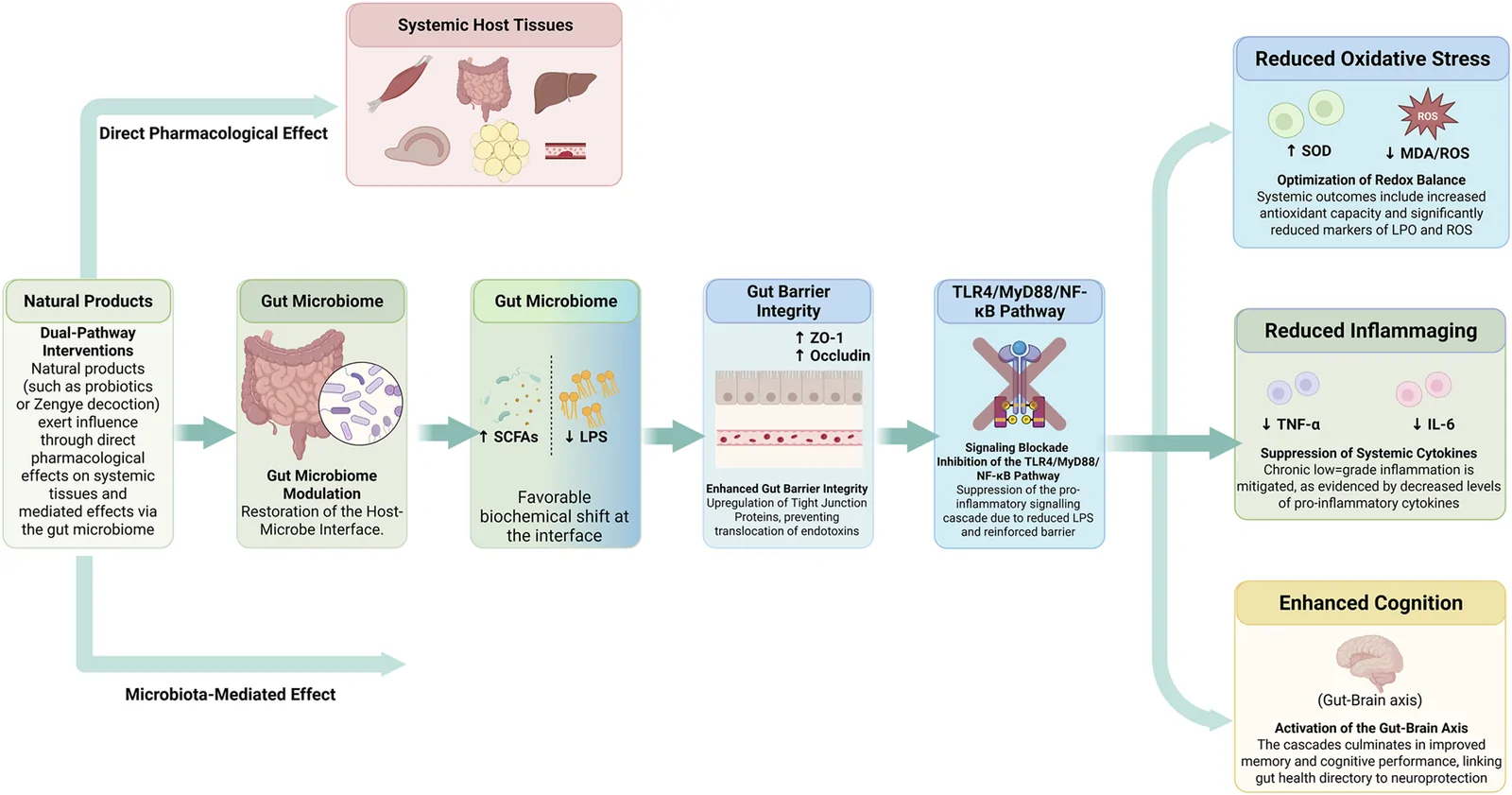
Schematic summary of how natural product interventions modulate gut microbiota composition and function, leading to increased short-chain fatty acid (SCFA) production and reduced lipopolysaccharide (LPS) levels. These changes enhance gut barrier integrity through the upregulation of tight junction proteins and suppression of TLR4/MyD88/NF-κB signalling. Collectively, these effects contribute to reduced oxidative stress, attenuation of inflammaging, and improved cognitive function, thereby supporting healthy ageing and systemic homeostasis.

In conclusion, the current preclinical evidence suggests that natural product interventions have the potential to modulate the gut microbiota, a shift that strongly correlates with reductions in age-related oxidative stress and systemic inflammation. However, the existing literature is predominantly restricted to short-term, small-scale rodent models and largely lacks rigorous causal validation using germ-free paradigms or direct metabolomics. Furthermore, previously hypothesized mechanisms linking these interventions to the gut-brain axis and the protection of genomic stability currently lack empirical substantiation. Therefore, while natural products represent a highly promising exploratory avenue for mitigating age-related dysbiosis, these preclinical findings must be interpreted with caution. Substantial translational hurdles, including anatomical differences, rigorous allometric dose scaling, and the profound environmental variance of the human microbiome, must be overcome through highly powered, personalized clinical trials before these interventions can be definitively recommended for human ageing. This is summarized in Figure 3.

## Data Availability

The original contributions presented in the study are included in the article/Supplementary Material, further inquiries can be directed to the corresponding author.
