# Supplementary material for: Natural products as modulators of age-associated gut microbiota dysbiosis: a systematic review of animal models
Source: Front Pharmacol. 2026 Aug 21;17:1809547. doi: 10.3389/fphar.2026.1809547 (PMC13541742; doi:10.3389/fphar.2026.1809547)
Supplement: Supplementary file 1 [file DataSheet1.pdf]

Supplementary Table 1: Detailed search strategy for each database.

Database	Search String
Web of Science	elderly OR aged (All Fields) and gut microbiota OR gut microbiome* OR gastrointestinal microbiome* OR gut flora OR gut microflora OR gut microbiota OR intestinal micro* OR gastric microbiome* (All Fields) and natural product* OR biologic* product* OR medicinal plant* OR medicinal herb* OR pharmaceutical plant* (All Fields) and animal stud* OR animal model* (All Fields)
Ovid MEDLINE	(AllFields:elderly OR aged) AND (AllFields:gut microbiota OR gut microbiome* OR gastrointestinal microbiome* OR gut flora OR gut microflora OR gut microbiota OR intestinal micro* OR gastric microbiome*) AND (AllFields:natural product* OR biologic* product* OR medicinal plant* OR medicinal herb* OR pharmaceutical plant*) AND (AllFields:animal stud* OR animal model*)
PubMed	(((elderly OR aged) AND (gut microbiota OR gut microbiome* OR gastrointestinal microbiome* OR gut flora OR gut microflora OR gut microbiota OR intestinal micro* OR gastric microbiome*)) AND (natural product* OR biologic* product* OR medicinal plant* OR medicinal herb* OR pharmaceutical plant*)) AND (animal stud* OR animal model*)
Scopus	(ALL (elderly OR aged) AND ALL (gut microbiota OR gut microbiome* OR gastrointestinal microbiome* OR gut flora OR gut microflora OR gut microbiota OR intestinal micro* OR gastric microbiome*) AND ALL (natural product* OR biologic* product* OR medicinal plant* OR medicinal herb* OR pharmaceutical plant*) AND ALL (animal stud* OR animal model*))

Supplementary Table 2: Taxonomic validation and reporting quality of plant-derived natural products included in the systematic review according to the ethnopharmacological and ConPhyMP reporting guidelines.

Author (Year)	Plant-based Interventions	Reported name	Validated Name	Family	Plant part	Preparation / Extraction method	Phytochemical characterization
He, Zhang et al. (2019)	Rapeseed peptide Maillard reaction products (MRPs)	Rapeseed (derived from defatted rapeseed meal)	*Brassica napus* L. [Brassicaceae] peptide MRPs	Brassicaceae	Seed	Defatted rapeseed meal undergoes enzymatic hydrolysis, lyophilized and followed by the Maillard reaction	N/A
Liu, Lin et al. (2019)	Zengye Decoction (ZYD)	*Radix scrophulariae*, *Radix rehmannia*, and *Ophiopogon japonicus*	*Scrophularia buergeriana* Miq. [Scrophulariaceae], *Rehmannia glutinosa* (Gaertn.) Libosch. ex DC. [Orobanchaceae], and *Ophiopogon japonicus* (Thunb.) Ker Gawl. [Asparagaceae]	Multiple families<br><br>(Scrophulariaceae, Orobanchaceae, Asparagaceae)	Dried root	The decoction consisted of 30g *Radix scrophulariae*, 24g of *Radix rehmannia* and 24g of *Ophiopogon japonicus*.<br><br>Primary extraction: Soaked in water for 4h (8:1 v/w), boiled for 1h and filtered.<br><br>Secondary extraction: Drugs re-boiled in water for 1h (6:1 v/w).<br><br>Filtrates then combined and concentrated to 1 g/mL.	High-performance Liquid Chromatography (HPLC)

Ren, Li et al. (2022)	*Arenga pinnata* flour (APF), *Arenga pinnata* starch (APS) and *Arenga pinnata* retrograded starch (APRS)	*Arenga pinnata* / Sugar palm	*Arenga pinnata* (Wurmb) Merr. [Arecaceae]	Arecaceae	Pulp	APF: Prepared directly from the pulp<br><br>APS: Isolated from the APF<br><br>APRS: Synthesised from APS via retrogradation	Composition analysis (Total starch, protein, fat, fiber, moisture and resistant starch percentage quantification)
Shenhua, Ziqin et al. (2020)	Fufang Zhenzhu Tiao Zhi (FZT)	*Fructus Ligustri* Lucidi, *Rhizoma coptidis*, *Herba cirsii* japonici, *Radix Salvia* miltiorrhiza, *Cortex Eucommiae*, *Radix Notoginseng*, *Radix Atractylodes* macrocephala and *Fructus Citri* Sarcodactylis	*Ligustrum lucidum* W.T.Aiton [Oleaceae], *Coptis chinensis* Franch. [Ranunculaceae], *Cirsium japonicum* DC. [Asteraceae], *Salvia miltiorrhiza* Bunge [Lamiaceae], *Eucommia ulmoides* Oliv. [Eucommiaceae], *Panax notoginseng* (Burkill) F.H.Chen [Araliaceae], *Atractylodes macrocephala* Koidz. [Asteraceae], and *Citrus × limon* (L.) Osbeck [Rutaceae].	Multiple families<br><br>(Oleaceae, Ranunculaceae, Asteraceae, Lamiaceae, Eucommiaceae, Araliaceae, Asteraceae, Rutaceae)	Fruit (*Fructus)*<br><br>Rhizome (*Rhizoma)*<br><br>Root (*Radix*)<br><br>Bark (*Cortex*)<br><br>Whole plant (*Herba*)	N/A<br><br>**The authors cited an external reference of their previously published study for the decoction preparation*	N/A
Xiang, Sun et al. (2023)	Guanshan cherry blossom extract (CBE)	Guanshan cherry blossom / *Crasus*	Prunus × lannesiana f. sekiyama (Koidz.) H.Hara [Rosaceae]	Rosaceae	Flower	Cherry blossom powder was mixed with distilled water (1:100 g/mL) and	UV-Vis Spectrophotometry (Quantification

		serrulate 'Sekiyama'				incubated overnight under constant shaking (100 rpm) at 25 °C. Mixture was then filtered and vacuum evaporated. The samples were then freeze-dried at -55 °C and sieved to obtain CBE powder.	of polysaccharides, total polyphenols, and total flavonoids)<br><br>Dumas Combustion: Protein quantification.<br><br>HPLC: Identification and relative quantification of specific polyphenols and flavonoids
Zhang, Chen et al. (2021)	Wushen (WS)<br><br>**a complex 55-ingredient food mixture (incorporating plants, fungi, and animal products)*	Wushen (WS)<br><br>**The authors highlighted some specific plant-based components: Grape (Vitis vinifera), Tartary buckwheat (Fagopyrum tataricum), Bilberries (Vaccinium myrtillus L.)*	Vitis vinifera L. [Vitaceae], Fagopyrum tataricum (L.) Gaertn. [Polygonaceae], Vaccinium myrtillus L. [Ericaceae]<br><br>**Highlighted components only*	Multiple families<br><br>(Vitaceae, Polygonaceae, Ericaceae)	N/A	55 ingredients (containing fruits, vegetables, grains, aquatic products, cooked meats, eggs, milk) were pulverized into powder without additional additives. These mixtures were then directly incorporated into standardized murine diet.	N/A
Chen, Zhang	Tangshen formula (TSF)	Tangshen formula (TSF)	N/A	N/A	N/A	N/A	N/A

et al. 2024)			** Composition not explicitly provided in the primary article; authors referred to an earlier publication for formulation details*				
Gao, Ji et al. (2024)	Ginsenoside Rg2	*Panax ginseng* C.A. Meyer	*Panax ginseng* C.A.Mey. [Araliaceae]	Araliaceae	N/A	Produced via the alkaline conversion reaction of primary ginsenoside Re.<br><br>** Detailed preparation parameters are cited via external references, not in-text*	HPLC<br><br>Liquid Chromatography /Electrospray Ionization Mass Spectrometry (LC/ESI-MS)

Abbreviations: N/A=not reported; HPLC=High-performance Liquid Chromatography
